# CW-fDOT with a standard optical brain model: improving hemodynamic sensitivity and functional localization

**DOI:** 10.1117/1.NPh.13.3.035011

**Published:** 2026-09-22

**Authors:** Tong Zhang, Xiaomeng Wang, Yuke Wang, Baole Wang, Limin Zhang, Feng Gao, Dongyuan Liu

**Affiliations:** a Tianjin University, College of Precision Instruments and Optoelectronics Engineering, Tianjin, China; b Tianjin University, State Key Laboratory of Precision Measuring Technology and Instruments, Tianjin, China; c Tianjin University, School of Artificial Intelligence, Tianjin, China

**Keywords:** continuous-wave functional diffuse optical tomography, standard optical brain, in vivo optical properties, magnetic resonance imaging, three-dimensional imaging

## Abstract

**Significance:**

Continuous-wave functional diffuse optical tomography (CW-fDOT) enables three-dimensional (3D) functional brain imaging using portable functional near-infrared spectroscopy systems. However, its reconstruction accuracy is often limited by simplified background models and empirical initialization of optical properties. Although subject-specific anatomical structures and optical properties can provide accurate prior information, the additional data-acquisition burden and workflow complexity limit their practical translation. Therefore, a reusable standardized model is urgently needed to improve CW-fDOT reconstruction performance while preserving its portability and practical applicability.

**Aim:**

This study aimed to develop and validate a CW-fDOT reconstruction framework based on a standard optical brain (SOB) model to improve cerebral hemodynamic reconstruction accuracy, activation detection, and functional brain-state discrimination without requiring additional acquisition of subject-specific anatomical structures or optical properties.

**Approach:**

Frontal anatomical statistics derived from magnetic resonance imaging and tissue-specific optical properties estimated by time-domain DOT were integrated to construct a frontal SOB model. The model can be scaled according to individual external head dimensions and incorporated into a source-detector distance-dependent CW-fDOT reconstruction strategy, thereby providing anatomical constraints and tissue-specific optical-property initialization for reconstruction. The framework was systematically validated through SOB model performance evaluation, numerical simulations, phantom experiments, and *in vivo* breath-holding experiments involving 48 subjects.

**Results:**

The SOB model preserved the major frontal tissue structures, physiologically reasonable optical properties, and the main photon-transport characteristics of subject-specific models. Numerical simulations showed that SOB initialization achieved the best reconstruction performance among the four initialization strategies. Phantom experiments further confirmed that, under realistic measurement conditions, the proposed method improved target localization, morphological recovery, and quantitative reconstruction. *In vivo* breath-holding experiments showed that, compared with homogeneous-background empirical initialization, SOB initialization identified more activated regions, with the number of significantly activated gray matter nodes increased by 79.52%. The extracted features also more effectively discriminated different functional brain states. Dynamic 3D imaging further showed that the main GM response region exhibited a 15.42% relative increase in mean oxyhemoglobin concentration change during the breath-holding period compared with the first 30-s resting period of one trial.

**Conclusions:**

Incorporating a reusable SOB model into CW-fDOT reconstruction provides effective anatomical constraints and optimized optical initialization, thereby improving quantitative reconstruction accuracy, activation detection, and functional brain-state discrimination. This framework offers a feasible pathway toward high-precision portable functional brain imaging, although its generalizability and clinical applicability require further validation in larger and more diverse populations.

## 1 Introduction

Functional near-infrared spectroscopy (fNIRS) is an established and increasingly used neuroimaging technique that combines portability, noninvasiveness, a favorable balance between spatial and temporal resolution, and high ecological validity. It has therefore been widely applied in cognitive neuroscience, psychiatric assessment, and neurodevelopmental evaluation.^1^^–^^5^ Among various fNIRS techniques, continuous-wave (CW) systems are the most commonly used because of their low cost, compact configuration, ease of integration, and suitability for wearable applications.^6^^–^^13^ CW-fNIRS mainly includes two imaging modalities: functional optical topography and functional diffuse optical tomography (fDOT). Optical topography is operationally simple, fast, and robust, making it suitable for routine brain-function assessment and monitoring.^14^ In contrast, CW-fDOT requires more complex system configurations and image reconstruction algorithms, but it enables three-dimensional (3D) functional imaging, provides depth discrimination, and is sensitive to absorption changes. Therefore, CW-fDOT has greater potential for precise brain-function assessment, mechanistic studies of cerebral hemodynamics, and clinical translation.^15^^–^^19^

Nevertheless, fDOT faces inherent practical challenges. CW-fDOT reconstructs the high-dimensional distribution of tissue optical properties from limited boundary light-intensity measurements, making it an inherently ill-posed inverse problem.^20^^–^^22^ To improve inversion stability, existing methods commonly introduce simplified layered geometries or incorporate subject-specific anatomical priors from structural imaging modalities such as magnetic resonance imaging (MRI) and computed tomography.^23^^–^^25^ In addition, the initialization of optical properties can markedly affect the convergence efficiency and reconstruction accuracy of the inverse algorithm. Inappropriate initial values may lead to slow convergence or convergence to local minima.^21^^,^^26^^,^^27^ Previous studies have shown that anatomically guided optical background models can improve the imaging sensitivity, spatial localization, and quantitative accuracy of CW-fDOT.^28^^–^^30^ Nevertheless, acquiring individualized structural information and absolute optical properties for each subject requires additional equipment, longer experimental time, and complex processing procedures, which partly compromises the inherent portability and application efficiency of CW-fDOT systems. Therefore, constructing a reusable standardized model with anatomical plausibility and optical-property support is important for improving the practicality and reconstruction reliability of CW-fDOT. To address these issues, this study proposes a quantitative CW-fDOT framework based on a standard optical brain (SOB) model. The framework first integrates MRI-derived frontal anatomical statistics with*in vivo* tissue optical properties measured by time-domain DOT (TD-DOT) to construct a SOB model. This model is then incorporated into CW-fDOT reconstruction to constrain tissue-layer structure and provide physiologically grounded initial optical parameters, thereby improving the stability and spatial accuracy of the inversion process.

In this framework, MRI and TD-DOT provide complementary priors for CW-fDOT reconstruction. MRI offers high spatial resolution and excellent soft-tissue contrast, making it suitable for identifying major brain tissue structures, including the scalp-skull (SS) layer, gray matter (GM), and white matter (WM).^31^^,^^32^ Optical-property measurement was performed using TD-DOT rather than CW measurements. Although CW systems are more convenient, they mainly record steady-state light-intensity changes and lack time-resolved information. In contrast, TD-DOT uses ultrashort pulsed light sources and time-resolved detectors to record the temporal point spread function, allowing extraction of photon time-of-flight (TOF) and other information closely related to tissue absorption and scattering.^33^ Therefore, this study integrated MRI-derived anatomical statistics with TD-DOT-measured tissue optical properties to construct the SOB model, which was then used as a reusable prior for CW-fDOT reconstruction. The proposed framework was systematically validated through numerical simulations, phantom experiments, and *in vivo* breath-holding experiments. Compared with conventional CW-fDOT methods based on empirical structural assumptions and fixed initial optical properties, the SOB-prior-based reconstruction method improved target localization, structural fidelity, and quantitative recovery. In addition, this method enhanced the extraction of task-evoked cerebral hemodynamic responses and improved activation detection sensitivity and brain-state classification performance. Overall, this study establishes a quantitative CW-fDOT reconstruction framework that balances modeling accuracy, experimental feasibility, and portability, providing a potential technical pathway for developing high-precision and generalizable functional near-infrared brain imaging methods.

## 2 Materials and Methods

**Fig. 1:**
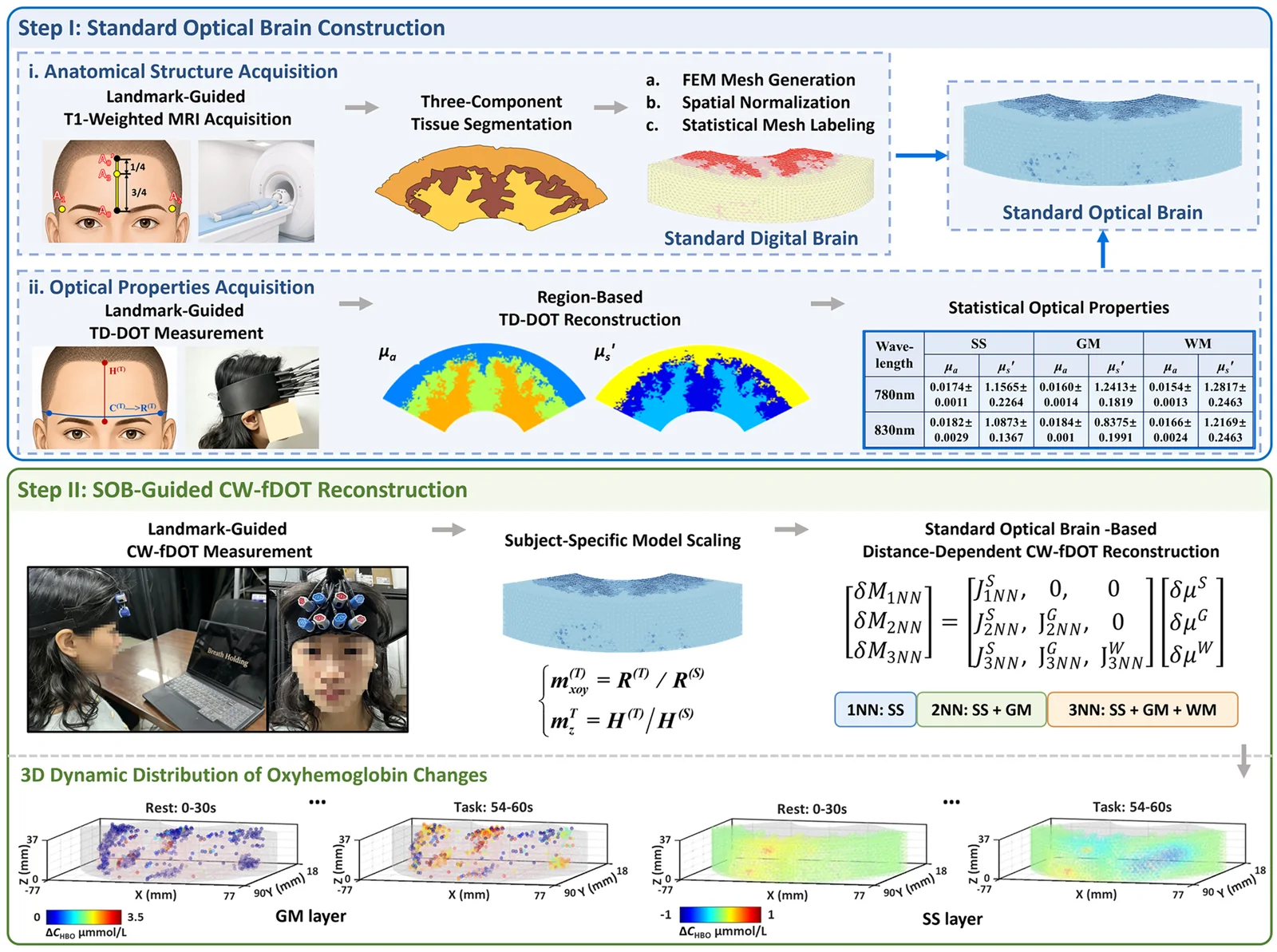
Overall workflow of the SOB-guided quantitative CW-fDOT framework. SOB, standard optical brain; CW-fDOT, continuous-wave functional diffuse optical tomography; MRI, magnetic resonance imaging; FEM, finite element method; TD-DOT, time-domain diffuse optical tomography; SS, scalp-skull; GM, gray matter; WM, white matter; $\mu_{a}$, absorption coefficient; $\mu_{s}^{\prime }$, reduced scattering coefficient; ${\Delta C}_{\mathrm{HbO}}$, oxyhemoglobin concentration change.

Figure 1 illustrates the overall workflow of the proposed framework, which consists of two stages: SOB model construction and its application to CW-fDOT reconstruction. First, frontal anatomical information was obtained using MRI, and a standard digital brain (DB) was constructed through tissue segmentation, spatial normalization, and statistical analysis of tissue-specific anatomical features. Meanwhile, *in vivo* tissue optical properties were measured using TD-DOT and integrated with the standard DB to generate the SOB model. The SOB model was then incorporated into CW-fDOT reconstruction and scaled according to the head dimensions of each target participant to generate a subject-adapted reconstruction model, thereby providing anatomical and optical priors for reconstruction. During reconstruction, source-detector distance-dependent measurement information was further incorporated to constrain the reconstruction contributions of different tissue layers and improve the 3D recovery of task-evoked hemodynamic changes.

### 2.1 Participants and Ethical Approval

This study included two cohorts of healthy right-handed participants. All participants had no history of neurological or psychiatric disorders and provided written informed consent. The study protocol was approved by the Ethics Committee of Tianjin University (TJUE-2025-226). The SOB construction cohort consisted of eight healthy participants (four males and four females; mean age, 25.88±1.27 years) and was used for MRI-based anatomical modeling and TD-DOT-based estimation of tissue optical properties. The independent CW-fDOT validation cohort consisted of 48 healthy participants (24 males and 24 females; mean age, 25.75±1.67 years). Participants in the validation cohort did not undergo individual MRI scanning or TD-DOT-based optical-property reconstruction, allowing the proposed SOB-guided reconstruction strategy to be evaluated under conditions without individualized anatomical or optical priors.

### 2.2 MRI and TD-DOT Guided Standard Optical Brain Model Construction

#### 2.2.1 MRI data acquisition and tissue segmentation

**Fig. 2:**
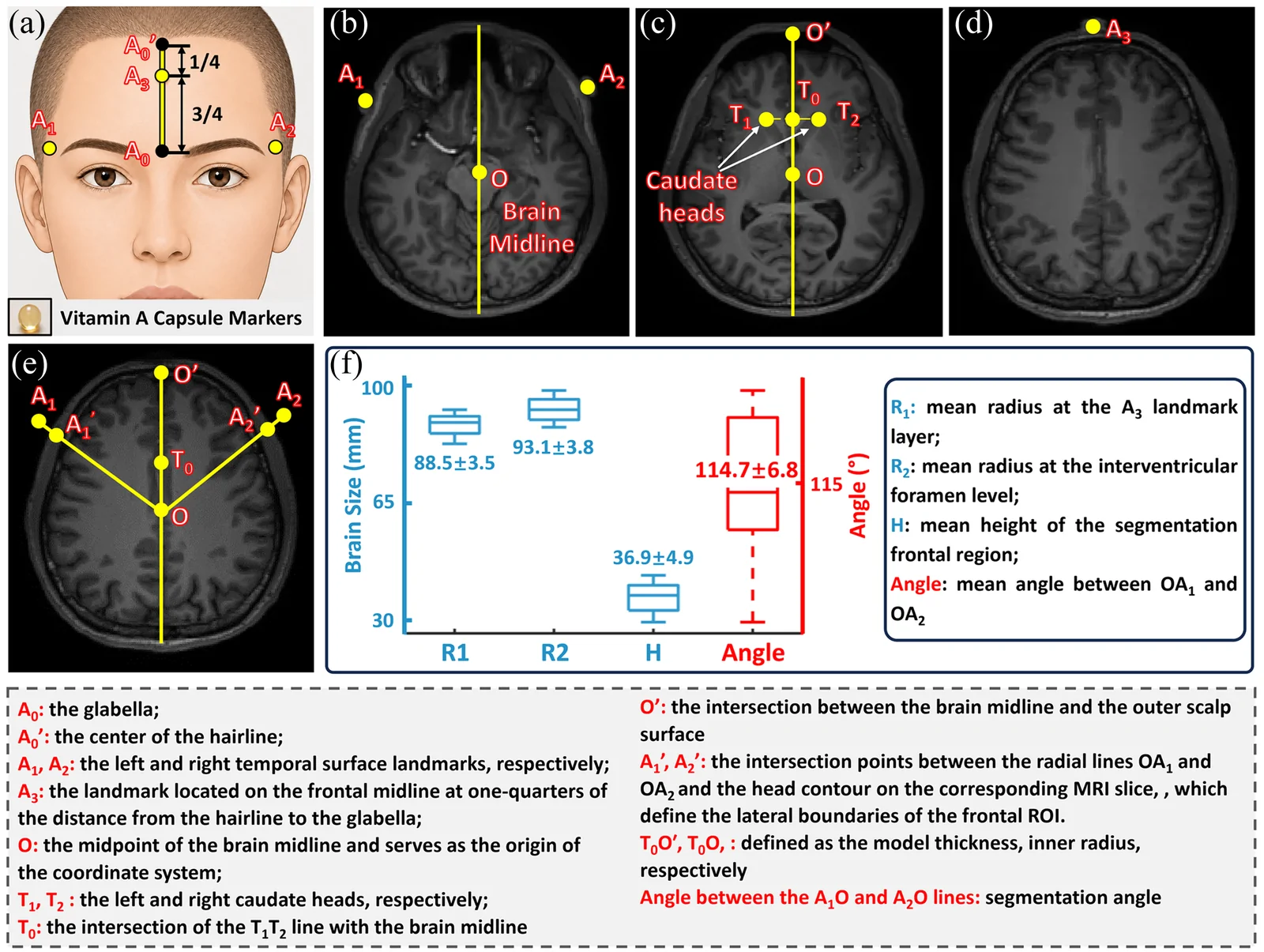
Anatomical landmarking, tissue segmentation, and head dimension statistics. (a) Placement of vitamin A capsules as MRI-visible fiducial markers at $A_{1}$, $A_{2}$ and $A_{3}$. (b) Axial slice containing markers $A_{1}$ and $A_{2}$. (c) Starting layer defined at the caudate-head level. (d) Ending layer identified by the first appearance of marker $A_{3}$. (e) Representative intermediate layer showing the definition of frontal-region boundaries and tissue segmentation. (f) Statistics of head dimensions used for standard DB model construction.

Because brain tissue morphology is highly similar across different cranial transverse sections, relying solely on intracranial anatomical structures makes it difficult to define the frontal modeling region in a stable and intuitive manner. In contrast, external anatomical landmarks provide clearer and more reproducible spatial references, reducing operator-dependent subjectivity and improving the consistency of region of interest (ROI) definition and tissue segmentation. Therefore, before MRI acquisition, three vitamin A (VA) capsules with a diameter of 4 mm were placed as external anatomical landmarks, as shown in Fig. 2(a). These capsules appeared as high-intensity markers on T1-weighted MRI images. Two capsules were placed at the bilateral temples ($A_{1}$ and $A_{2}$) to define the left and right boundaries of the frontal modeling region, whereas the third capsule was placed on the frontal midline at one quarter of the distance from the hairline to the glabella ($A_{3}$) to determine the terminal slice for tissue segmentation. These landmarks also served as common spatial references for TD-DOT optical-property measurements and CW-fDOT functional measurements, thereby enhancing the spatial correspondence between multimodal measurements. High-resolution whole-brain 3D T1-weighted images were acquired using a 3.0T MRI scanner (Philips Ingenia Elition S) and imported into Amira software (Visage Imaging) for tissue segmentation and tetrahedral meshing.^34^

According to the requirements of fNIRS imaging and the optical properties of head tissues, ^35^^–^^41^ the arachnoid mater, cerebrospinal fluid, scalp, and skull were merged into a single SS layer, whereas GM and WM were defined as separate layers. A coordinate system was established using the midpoint of the brain midline (O) as the origin. Anatomical landmarks, including $A_{1}$ and $A_{2}$ [Fig. 2(b)], $T_{1}$ and $T_{2}$ representing the left and right caudate heads, and $T_{0}$ representing the intersection of the $T_{1}T_{2}$ line with the brain midline, were identified [Fig. 2(c)]. The slice containing the caudate nuclei was defined as the starting layer, whereas the first slice showing the $A_{3}$ landmark was defined as the ending layer [Fig. 2(d)]. The model thickness was defined as $T_{0}O^{\prime }$, where $O^{\prime }$ denotes the intersection between the brain midline and the outer scalp surface. The inner radius was defined as $T_{0}O$, and the segmentation angle was determined by the angle between the $A_{1}O$ and $A_{2}O$ lines [Fig. 2(e)]. $A_{1}^{\prime }$ and $A_{2}^{\prime }$ denote the corresponding intersection points between the ${\mathrm{OA}}_{1}$ and ${\mathrm{OA}}_{2}$ radial lines and the head contour on the MRI slice, which were used to define the lateral boundaries of the frontal region. Because the grayscale intensities of the scalp and GM layers were relatively similar, manual segmentation was performed in this study. Within the defined frontal region, tissue boundaries were delineated slice by slice based on grayscale contrast, anatomical boundary features, and structural continuity between adjacent slices. The accuracy of tissue boundaries and inter-layer continuity were then inspected to avoid discontinuities or abnormal overlaps between tissue layers. Finally, 3D models were generated from the confirmed segmentation results, and volumetric meshing was performed to obtain the original DB model.

#### 2.2.2 Model normalization and statistical mesh labeling

To eliminate inter-subject head-size variability in the statistical analysis of the original DB mesh nodes, all original DBs were standardized. As shown in Fig. 2(f), head dimension measurements from the eight participants indicated similar radii between the first and last segmented layers (at the caudate nuclei slice: 93.1±3.8  mm; mean radius at the $A_{3}$ landmark layer: 88.5±3.5  mm). Consequently, a standardized brain model (SBM) with identical upper and lower surface dimensions was defined with the following parameters: outer radius = 90 mm (mean of the starting and ending layer radii), inner radius = 35 mm (mean length of $T_{0}O$), height = 37 mm (mean total segmentation height), and central angle = 115 deg (derived from the segmented region proportion).

**Fig. 3:**
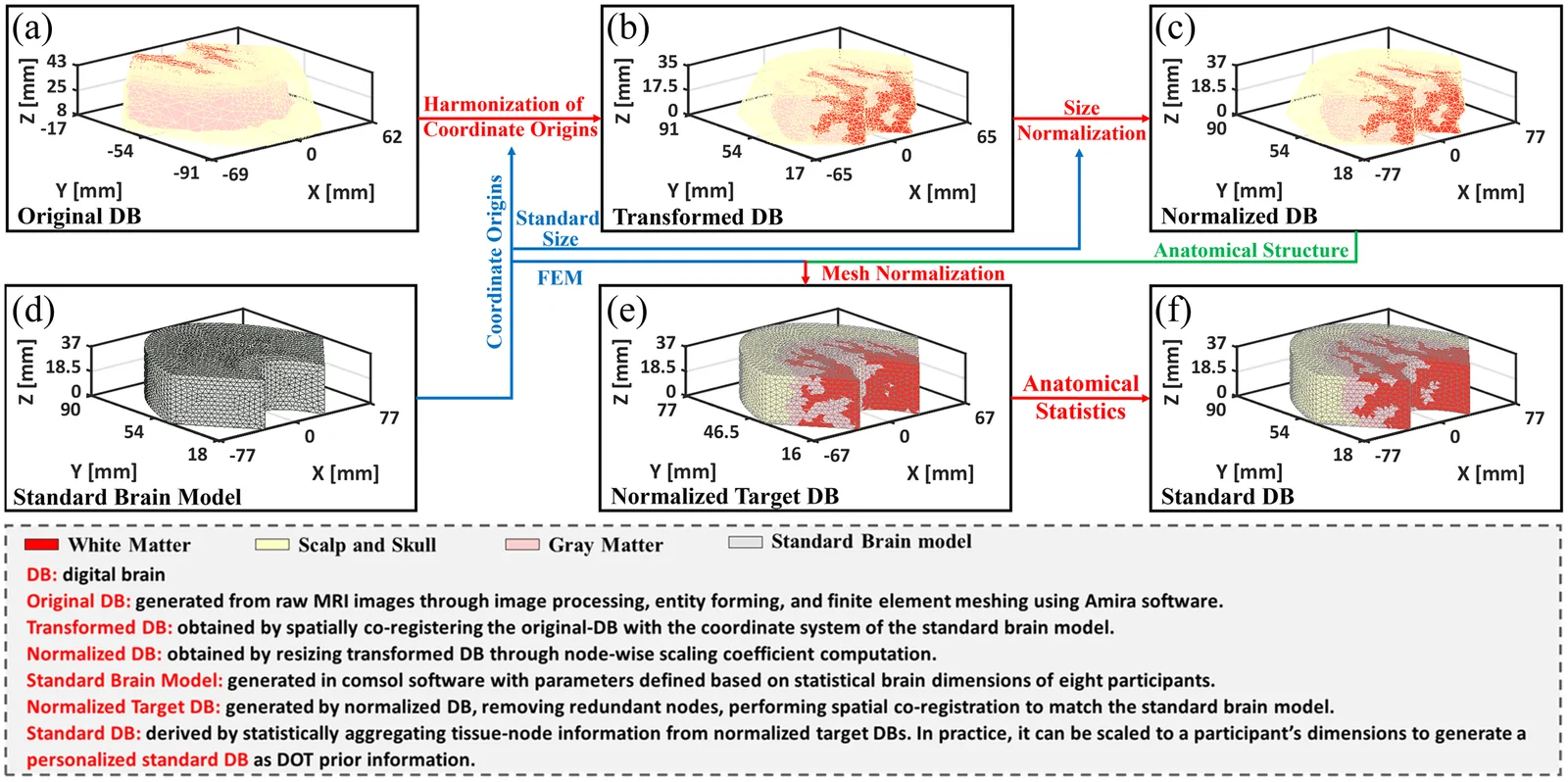
Workflow for constructing the standard digital brain model. (a) Original DB. (b) Transformed DB. (c) Normalized DB. (d) Standard brain model. (e) Normalized target DB. (f) Standard DB.

The standardization workflow is illustrated in Fig. 3. The detailed methodology follows our prior work,^34^ and this section presents the key steps. First, all original DBs [Fig. 3(a)] were transformed into the SBM coordinate system to obtain transformed DB [Fig. 3(b)]. Next, scaling coefficients in the XOY plane $m_{\mathrm{xoy}}=R^{\left(O\right)}/R^{\left(S\right)}$ and along the Z-axis $m_{z}=H^{\left(O\right)}/H^{\left(S\right)}$ were calculated based on individual head circumference and segmentation height ($H^{\left(O\right)}$) to generate normalized DB [Fig. 3(c)]. Normalized DB was then registered to the SBM mesh (16,278 nodes) [Fig. 3(d)], and nearest-node distance matching was applied to form the normalized target DB [Fig. 3(e)]. Finally, WM and GM node assignments were determined using the maximum probability method, while SS using the coordinate averaging methodology,^34^ resulting in the standard DB [Fig. 3(f)] containing statistical tissue-type information.

$$\begin{array}{c} F(r_{n}^{\left(S\right)},C_{n}^{\left(S\right)},\Omega_{e}^{\left(S\right)}):\left\{ \begin{array}{l} r_{n}^{\left(S\right)}=[x_{n}^{\left(S\right)},y_{n}^{\left(S\right)},z_{n}^{\left(S\right)}],n=1,2,\ldots ,N^{\left(S\right)}\\ C_{n}^{\left(S\right)}=c,c\in \{1,2,\ldots ,C\}\\ \Omega_{e}^{\left(S\right)}=[i_{e},j_{e},k_{e},l_{e}],i_{e},j_{e},k_{e},l_{e}\in \{1,2,\ldots ,N^{\left(S\right)}\} \end{array}\right., \end{array}$$ (1)

where S denotes the standard type, $r_{n}^{\left(S\right)}$ is the spatial coordinate of the n’th node, $C_{n}^{\left(S\right)}$ is the n’th node’s tissue type (c=1–3 represents, SS, GM, and WM, respectively), $\Omega_{e}^{\left(S\right)}$ is the e’th tetrahedral element, and $i_{e},j_{e},k_{e}$ and $l_{e}$ are the corresponding nodes.

#### 2.2.3 Optical properties acquisition using TD-DOT

*In vivo* optical properties of different brain tissues were estimated by TD-DOT based on the generalized pulse spectrum technique,^42^ in which calibrated temporal signals were transformed into the complex frequency domain by Laplace transform and used for iterative inversion. To reduce system-related effects, including the instrument response function and source-detector coupling variations, target measurements were calibrated using data acquired from a size-matched reference phantom under the same protocol.^34^^,^^43^ Considering the limited measurements and ill-posed nature of TD-DOT reconstruction, optical properties were assumed to be homogeneous within each anatomical tissue region of the personalized DB model. Accordingly, voxel-wise reconstruction was reduced to region-wise estimation by transforming the voxel-wise Jacobian matrix into a region-wise form:^44^

$$\overset{˜}{J}=J\mathrm{G,}$$ (2)

where $\overset{˜}{J}$, J, and G denote the region-wise Jacobian matrix, the original voxel-wise Jacobian matrix, and the tissue-region-to-voxel mapping matrix,^43^^,^^45^^–^^47^ respectively. Each column of G represents an anatomical tissue region and defines the assignment of voxels to that region.

For each participant, the mean absorption coefficient ($\mu_{a}$) and reduced scattering coefficient ($\mu_{s}^{\prime }$) were reconstructed for each tissue region and wavelength. To generate standardized optical priors for the SOB model, the reconstructed tissue-specific optical properties were then averaged across all participants involved in SOB construction for the same tissue type and wavelength. The resulting averaged optical properties were assigned to the corresponding anatomical layers of the standard DB model, thereby generating the SOB model. This model can be represented as a finite element model containing anatomical structure (tissue labels) and wavelength-dependent optical properties:

$$F^{\mathrm{OB}}(\lambda )=\{r_{n}^{\left(S\right)},C_{n}^{\left(S\right)},\Omega_{e}^{\left(S\right)},{\overset{¯}{\theta }}_{n}^{\mathrm{OB}}(\lambda )\},$$ (3)

where ${\overset{¯}{\theta }}_{C_{n}}^{\mathrm{OB}}(\lambda )$ denotes the standardized optical-property vector assigned to node n at wavelength λ, according to its tissue label. Details of the TD-DOT procedure used to obtain these tissue-specific optical properties, including the reconstruction algorithm and system configuration, are described in Secs. S1 and S2 of the Supplementary Material.

#### 2.2.4 Performance evaluation of the SOB model

The generalizability of the SOB model was evaluated from three complementary perspectives: anatomical fidelity, optical-property plausibility, and photon-transport consistency. Anatomical fidelity was quantified by calculating the proportion of nonoverlapping nodes between the standardized DB model and each original DB model. The plausibility of the optical properties was assessed by comparing the $\mu_{a}$ and $\mu_{s}^{\prime }$of different tissue layers estimated by TD-DOT with the ranges reported in the literature. Photon-transport consistency was evaluated by comparing photon propagation characteristics between the standardized DB model and the individual original DB models, using MonteCarlo (MC) simulations implemented with the open-source mesh-based MC software MMC.^48^^,^^49^

**Fig. 4:**
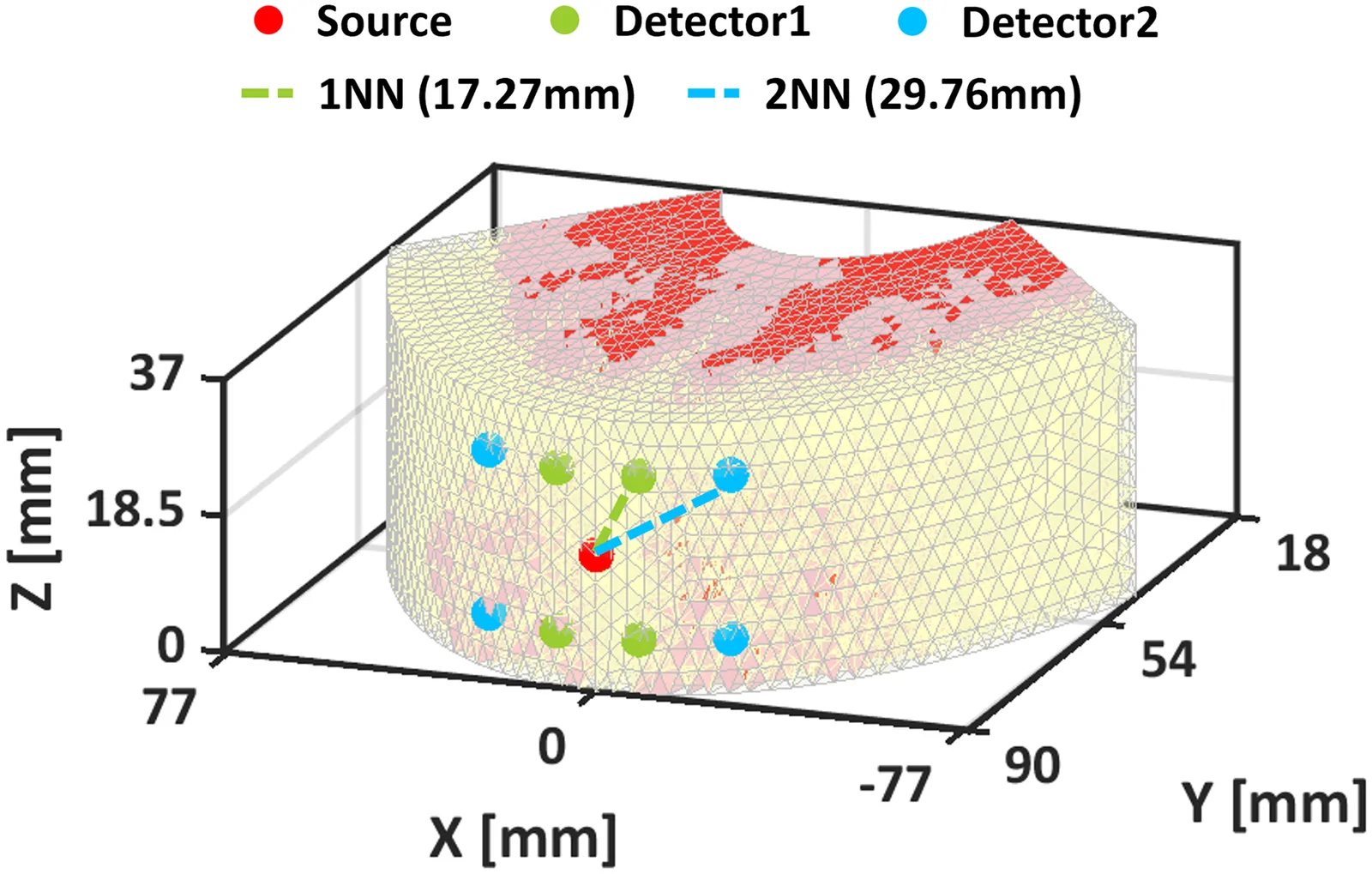
Numerical simulation model and source-detector array configuration for photon-transport consistency evaluation.

MC simulation was selected because it can flexibly handle finite-size 3D boundaries, layered tissue optical properties, realistic source-detector configurations, and localized absorption perturbations. Detailed simulation parameters are provided in Table S1 of the Supplementary Material, and the source-detector array configuration is shown in Fig. 4. The light source was placed at the center of the model, and the detectors were symmetrically arranged, including four first-nearest-neighbor (1NN) detectors and four second-nearest-neighbor (2NN) detectors, with source–detector distances of 17.27 and 29.76 mm, respectively. The mean temporal point spread function (TPSF) curves for each source-detector pair were recorded and quantitatively compared using the relative root mean square error (RRMSE), relative mean TOF error (RMTOF), relative full width at half maximum error (RFWHM), and Pearson correlation coefficient (r). These metrics characterize pointwise curve deviation, temporal shift, temporal broadening, and morphological consistency, respectively. Lower RRMSE, RMTOF, and RFWHM values, together with an r value closer to 1, indicate a higher similarity between TPSF curves.

$$\left\{ \begin{array}{l} {\mathrm{RRMSE}}_{i}=\frac{\sqrt{\frac{1}{T}\overset{T}{\underset{t=1}{\sum }}{\left[{\mathrm{TPSF}}_{S}(t)-{\mathrm{TPSF}}_{O_{i}}(t)\right]}^{2}}}{\frac{1}{T}\overset{T}{\underset{t=1}{\sum }}{\mathrm{TPSF}}_{O_{i}}\left(t\right)}\\ {\mathrm{RMTOF}}_{\mathrm{rel},i}=\frac{\left|{\mathrm{MTOF}}_{S}-{\mathrm{MTOF}}_{O_{i}}\right|}{{\mathrm{MTOF}}_{O_{i}}},\mathrm{MTOF}=\frac{\overset{T}{\underset{t=1}{\sum }}t\cdot \mathrm{TPSF}(t)}{\overset{T}{\underset{t=1}{\sum }}\mathrm{TPSF}(t)}\\ {\mathrm{RFWHM}}_{\mathrm{rel},i}=\frac{\left|{\mathrm{FWHM}}_{S}-{\mathrm{FWHM}}_{O_{i}}\right|}{{\mathrm{FWHM}}_{O_{i}}}\\ r_{i}=\frac{\overset{T}{\underset{t=1}{\sum }}({\mathrm{TPSF}}_{S}(t)-{\overset{¯}{\mathrm{TPSF}}}_{S})({\mathrm{TPSF}}_{O_{i}}(t)-{\overset{¯}{\mathrm{TPSF}}}_{O_{i}})}{\sqrt{\overset{T}{\underset{t=1}{\sum }}{\left[{\mathrm{TPSF}}_{S}(t)-{\overset{¯}{\mathrm{TPSF}}}_{S}\right]}^{2}}\sqrt{\overset{T}{\underset{t=1}{\sum }}{\left[{\mathrm{TPSF}}_{O_{i}}(t)-{\overset{¯}{\mathrm{TPSF}}}_{O_{i}}\right]}^{2}}} \end{array},\right.$$ (4)

where ${\mathrm{TPSF}}_{S}(t)$ and ${\mathrm{TPSF}}_{O_{i}}(t)$ denote the photon counts at time point t of the TPSF curves generated by MC simulations for the standard DB model and the i-th individual original DB model, respectively,; T denotes the number of time points, with a temporal sampling interval of 12.2 ps and T=410.

### 2.3 SOB-Guided Distance-Dependent CW-fDOT Reconstruction

#### 2.3.1 Theory

Although conventional CW-fDOT enables 3D brain function reconstruction, its reconstruction accuracy is often limited by simplified homogeneous models and empirical optical-property initialization.^50^^,^^51^ Incorporating subject-specific anatomical structures or prior information on the optical background can improve reconstruction accuracy,^23^^–^^25^ but this reduces the portability and operational efficiency required for CW-fDOT applications. To balance anatomical realism, optical prior information, and practical applicability, this study incorporated the SOB model into the CW-fDOT reconstruction framework, where it served as both an anatomical prior and an optical-property initialization basis.

For each subject, the SOB model was scaled according to individual frontal head dimensions to generate a personalized SOB model for reconstruction. The scaling factors in the XOY plane and along the Z-axis were calculated from the subject’s frontal radius $R_{\text{frontal}}$ and frontal height $H_{\text{frontal}}$ respectively, relative to those of the reference model ($R_{\mathrm{ref}}$, $H_{\mathrm{ref}}$): $s_{xy}=R_{\text{frontal}}/R_{\mathrm{ref}}$, $s_{z}=H_{\text{frontal}}/H_{\mathrm{ref}}$. This scaling procedure enabled subject-specific adaptation of the SOB model using external frontal head dimensions, without requiring an individual MRI image. The scaled SOB model retained the standardized tissue labels and wavelength-dependent tissue optical properties, thereby providing both anatomical and optical priors for CW-fDOT reconstruction.

To further exploit the depth-dependent sensitivity of CW-fDOT measurements, first-, second-, and third-nearest-neighbor (3NN) channels were incorporated into the reconstruction, based on the assumption that short-separation channels are primarily sensitive to superficial tissues, whereas longer-separation channels contain progressively greater contributions from deeper cortical tissues.^52^ Accordingly, a distance- dependent, layer-specific Jacobian model was formulated as follows:

$$\begin{array}{c} {}[\begin{array}{c} \delta M_{1\mathrm{NN}}\\ \delta M_{2\mathrm{NN}}\\ \delta M_{3\mathrm{NN}} \end{array}]=[\begin{array}{c} J_{1\mathrm{NN}}^{\mathrm{SS}},0,0\\ J_{2\mathrm{NN}}^{\mathrm{SS}},J_{2\mathrm{NN}}^{\mathrm{GM}},0\\ J_{3\mathrm{NN}}^{\mathrm{SS}},J_{3\mathrm{NN}}^{\mathrm{GM}},J_{3\mathrm{NN}}^{\mathrm{WM}} \end{array}][\begin{array}{c} \delta \mu^{\mathrm{SS}}\\ \delta \mu^{\mathrm{GM}}\\ \delta \mu^{\mathrm{WM}} \end{array}], \end{array}$$ (5)

where $\delta M_{1\mathrm{NN}}$, $\delta M_{2\mathrm{NN}}$, and $\delta M_{3\mathrm{NN}}$ represent the boundary light intensity changes for the 1NN, 2NN, and 3NN channels, respectively; $J_{1\mathrm{NN}}^{\mathrm{SS}}$, $J_{2\mathrm{NN}}^{\mathrm{SS}}$, $J_{3\mathrm{NN}}^{\mathrm{SS}}$, $J_{2\mathrm{NN}}^{\mathrm{GM}}$, $J_{3\mathrm{NN}}^{\mathrm{GM}}$, $J_{2\mathrm{NN}}^{\mathrm{GM}}$, and $J_{3\mathrm{NN}}^{\mathrm{WM}}$ denote the Jacobian matrices of the 1NN, 2NN, and 3NN channels for the SS, GM, and WM layers, respectively; $\delta \mu^{\mathrm{SS}}=[\delta \mu_{1}^{SS},\cdots ,\delta \mu_{NS}^{SS}]$, $\delta \mu^{\mathrm{GM}}=[\delta \mu_{1}^{GM},\cdots ,\delta \mu_{NG}^{GM}]$ and $\delta \mu^{\mathrm{WM}}=[\delta \mu_{1}^{WM},\cdots ,\delta \mu_{NW}^{WM}]$ represent the changes in $\mu_{a}$ at the segmented nodes of the SS, GM, and WM layers; NS, NG and NW indicate the number of nodes in the SS, GM, and WM layers, respectively. In this formulation, 1NN channels were assumed to mainly reflect SS-layer changes, 2NN channels were sensitive to both SS and GM layers, and 3NN channels contained contributions from SS, GM, and WM layers, i.e., $J_{1\mathrm{NN}}^{\mathrm{GM}}=0$, $J_{1\mathrm{NN}}^{\mathrm{WM}}=0$, and $J_{2\mathrm{NN}}^{\mathrm{WM}}=0$. Incorporating the dominant depth sensitivity of different source-detector separations helped reduce cross-layer interference.

#### 2.3.2 Data acquisition

**Fig. 5:**
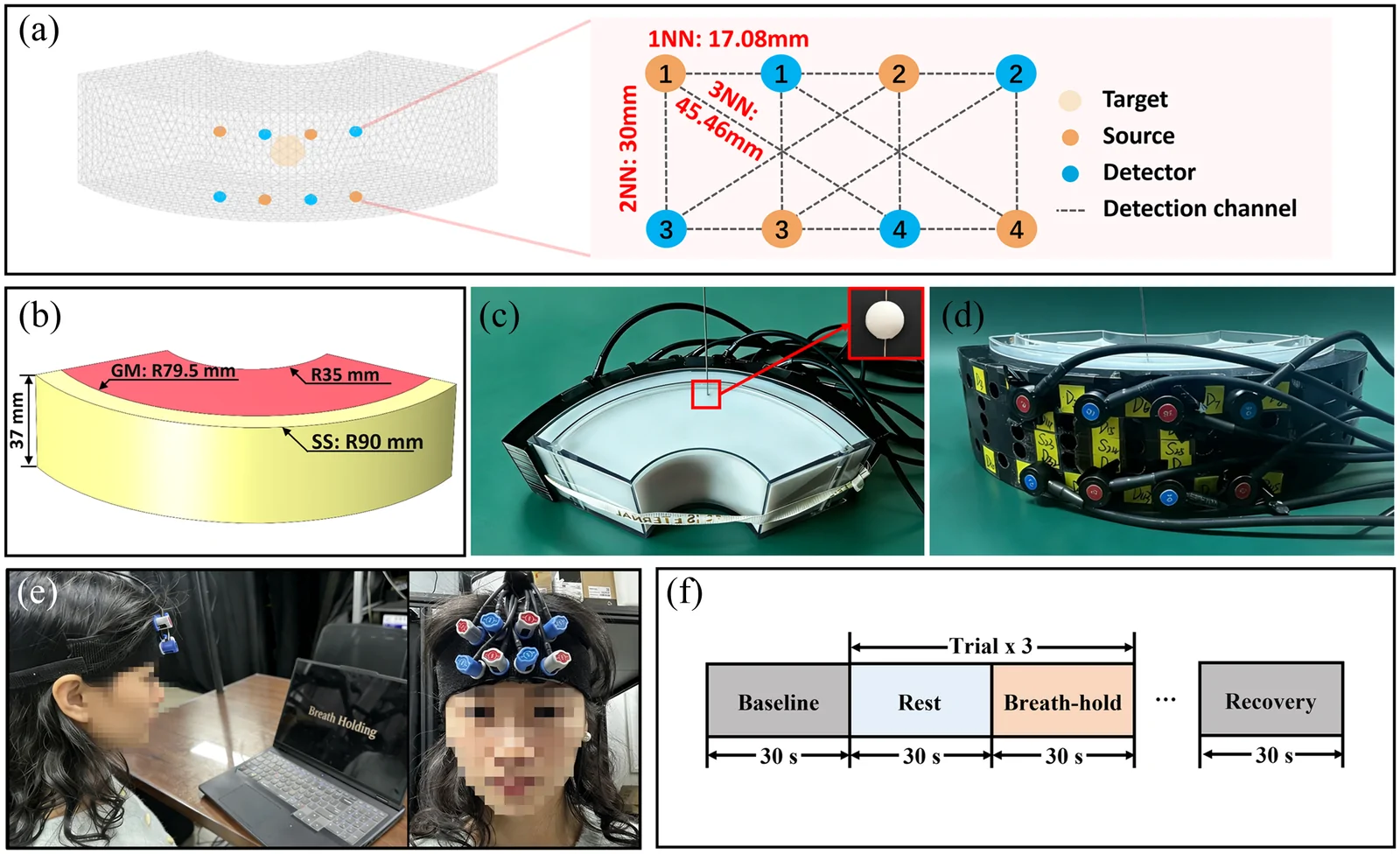
Simulation, phantom, and in vivo experimental configurations. (a) Single-target location, and 4-source/4-detector optode array with 1NN, 2NN, and 3NN channels. (b) SS/GM layer phantom geometry. (c) Physical phantom. (d) Source-detector configuration of CDMA-fNIRS system. (e) *In vivo* CW-fDOT data acquisition. (f) Breath-holding paradigm consisting of a 30 s baseline, three repeated 30 s rest/30 s breath-holding trials, and a 30 s recovery period.

CW-fDOT data were acquired using a self-developed portable fully parallel code-division multiple-access fNIRS (CDMA-fNIRS) system.^53^ The system provided dual-wavelength, multisource illumination and multidistance synchronous detection at 780 and 830 nm. The optode array used an interleaved source-detector configuration with four sources and four detectors [Fig. 5(a)], forming 14 measurement channels: six 1NN channels with a source-detector distance of 17.08 mm, four 2NN channels with a distance of 30 mm, and four 3NN channels with a distance of 45.46 mm. Data were sampled at 20 Hz to record task-evoked hemodynamic responses. Details of the hardware configuration, signal modulation, and detection procedures are provided in Section S4 of the Supplementary Material.

### 2.4 Experimental Validation of SOB-Guided Distance-Dependent CW-fDOT

#### 2.4.1 Numerical simulation design

Forward simulations were performed using a randomly selected original DB model, with tissue optical properties assigned based on the mean *in vivo* values estimated using TD-DOT. Reconstruction was then performed using the SOB model, thereby allowing a more objective evaluation of the generalizability of the SOB-based reconstruction framework. Both forward data generation and the forward modeling used in inverse reconstruction were implemented using MC simulations,^48^^,^^49^ with detailed simulation parameters listed in Table S1 of the Supplementary Material. As shown in Fig. 5(a), a spherical target with a radius of 7.5 mm was embedded in the forward model at a depth of 10.5 mm, with its center located at x=0  mm, y=72  mm, and z=18.5  mm, to mimic a localized brain activation region. To evaluate the effect of hemodynamic absorption changes on reconstruction performance, target-to-background absorption contrast ratios of 1.5, 2.0, and 3.0 were examined. An optimized overlapping source-detector array consisting of 1NN, 2NN, and 3NN channels was used to improve detection sensitivity.

To clarify whether the improvement achieved by SOB-based reconstruction primarily originated from the anatomical background model or from the initial optical properties, four initialization conditions were compared: (1) Homo-Emp. init., representing the conventional CW-fDOT model, used a homogeneous background model with empirical initial optical properties assigned to the entire head model, where $\mu_{a}=0.01\;{\mathrm{mm}}^{-1}$ and $\mu_{s}^{\prime }=1\;{\mathrm{mm}}^{-1}$; (2) Homo-TVFW init. retained the homogeneous background model but determined the initial optical properties using tissue-volume-fraction weighting (TVFW). The volume fractions of SS, GM, and WM were 25.79%, 30.35%, and 43.86%, respectively, yielding weighted initial values of $\mu_{a}=0.0172\;{\mathrm{mm}}^{-1}$ and $\mu_{s}^{\prime }=1.1297\;{\mathrm{mm}}^{-1}$; (3) SDB-Emp. init. used the standard DB anatomical background model with empirical initial optical properties, where $\mu_{a}=0.01\;{\mathrm{mm}}^{-1}$ and $\mu_{s}^{\prime }=1\;{\mathrm{mm}}^{-1}$; (4) SOB init., which combined the SDB anatomical background model with tissue-specific initial optical properties; the initial optical properties of the SS, GM, and WM layers were determined from tissue-averaged values reconstructed by TD-DOT at 780 and 830 nm. In addition, considering that real brain activation may involve multiple regions, a double-target simulation comparing Homo-Emp. init. and SOB init. is provided in Section S5 of the Supplementary Material to evaluate reconstruction performance under multi-region activation conditions.

#### 2.4.2 Phantom experiment design

The phantom experiment served as an intermediate validation step between numerical simulations and *in vivo* experiments. Compared with numerical simulations, it incorporated real optical measurements, system noise, and experimental uncertainties, thereby enabling evaluation of the practical robustness of the reconstruction method. Compared with *in vivo* experiments, it allowed quantitative assessment under conditions in which the target geometry, location, and absorption contrast were known. To ensure comparability, the target optical properties, absorption contrast, and source-detector array configuration in the phantom experiment were kept consistent with those used in the numerical simulations. Because the optical properties of GM and WM are relatively similar but differ markedly from those of the SS layer, GM and WM were approximated as a single brain-tissue layer. Accordingly, an SS/GM-equivalent two-layer phantom was designed, as shown in Fig. 5(b). This design preserved the major optical differences between superficial and cerebral tissues while reducing fabrication complexity and facilitating target embedding. As shown in Fig. 5(c), the phantom consisted of a liquid base and a solid spherical target. The liquid base was prepared using India ink, Intralipid, and distilled water, while the solid spherical target was fabricated using India ink, titanium dioxide, and silicone and fixed with a fine needle. The source–detector array configuration is shown in Fig. 5(d).

#### 2.4.3 In vivo breath-holding experiment design

Experiments were conducted in a quiet, dimly lit room. A custom flexible headband was used to secure the multichannel optode array over the participant’s forehead, as shown in Fig. 5(e). The array was positioned using the same external anatomical landmarks as those used for MRI-based modeling and TD-DOT measurements, with the lower edge aligned to the superior border of the eyebrows and the lateral ends placed near the bilateral temples. Before the experiment, each participant’s head circumference and forehead height were measured for subject-specific scaling of the SOB model, which was then used as the structural and optical prior for CW-fDOT reconstruction. A simple and repeatable breath-holding paradigm was used to induce hemodynamic changes in the prefrontal region. Previous studies^54^^–^^56^ have shown that controlled breath holding, dyspnea-related challenges, and apnea can activate the prefrontal cortex. Compared with cognitive tasks, the breath-holding task is easier to perform and requires less sustained attention from participants. As shown in Fig. 5(f), participants first underwent a 30 s eyes-closed resting baseline, followed by three alternating cycles of 30 s rest and 30 s breath holding, and finally a 30 s eyes-closed rest period. Participants were instructed to minimize head motion throughout the experiment. The paradigm was programmed using Psychtoolbox in MATLAB R2020a.

### 2.5 Data Processing, Statistical Analysis, and Performance Evaluation

#### 2.5.1 CW-fNIRS signal preprocessing and hemoglobin conversion

During data acquisition, the coefficient of variation (CV) of the raw light-intensity signal was calculated in real time for each channel. If the CV exceeded 10%, the headband position was adjusted to improve the optical coupling between the optodes and the scalp, and the light-source output power was adjusted when necessary. The measurement was then repeated until the CV values of all channels were ≤10%. Through this quality-control procedure, the complete set of 14 channels was retained for all participants, ensuring that subsequent reconstruction and statistical analysis were performed using a consistent source-detector layout and the same number of channels, thereby avoiding potential bias introduced by inconsistent channel configurations.

The raw CW-fNIRS light-intensity signals were then processed using a third-order band-pass filter with a frequency range of 0.05 to 0.20 Hz to suppress low-frequency drift and high-frequency physiological noise outside the task-related hemodynamic frequency band. The filtered dual-wavelength light-intensity signals were used to reconstruct $\mu_{a}$ changes at 780 and 830 nm using both the Homo-Emp. init. and SOB init. methods. The reconstructed dual-wavelength absorption changes were further converted into hemoglobin concentration changes according to the linear spectral absorption relationship.^1^^–^^3^ For each reconstructed node and time point, oxyhemoglobin and deoxyhemoglobin concentration changes were calculated by inverting the extinction-coefficient matrix:

$$\left[\begin{array}{c} \Delta \mu_{a}\left(780\right)\\ \Delta \mu_{a}\left(830\right) \end{array}\right]=\left[\begin{array}{cc} \varepsilon_{\mathrm{HbO}}\left(780\right) & \varepsilon_{\mathrm{HbR}}\left(780\right)\\ \varepsilon_{\mathrm{HbO}}\left(830\right) & \varepsilon_{\mathrm{HbR}}\left(830\right) \end{array}\right]\left[\begin{array}{c} \Delta C_{\mathrm{HbO}}\\ \Delta C_{\mathrm{HbR}} \end{array}\right],$$ (6)

where, $\varepsilon_{\mathrm{HbO}}$ and $\varepsilon_{\mathrm{HbR}}$ denote the wavelength-dependent molar extinction coefficients of oxyhemoglobin and deoxyhemoglobin, respectively. In this study, the extinction coefficients were set as $\varepsilon_{\mathrm{HbO}}(780)=0.71\;{\mathrm{mM}}^{-1}\cdot {\mathrm{cm}}^{-1}$, $\varepsilon_{\mathrm{HbO}}(830)=0.97\;{\mathrm{mM}}^{-1}\cdot {\mathrm{cm}}^{-1}$, $\varepsilon_{\mathrm{HbR}}(780)=1.08\;{\mathrm{mM}}^{-1}\cdot {\mathrm{cm}}^{-1}$, $\varepsilon_{\mathrm{HbR}}(830)=0.69\;{\mathrm{mM}}^{-1}\cdot {\mathrm{cm}}^{-1}$. The reconstructed oxyhemoglobin concentration change (${\Delta C}_{\mathrm{HbO}}$) was used for subsequent functional analysis.

#### 2.5.2 GLM-based GM-layer activation analysis

Because this study focused on cortical hemodynamic responses induced by the breath-holding task, the activation analysis was performed on the GM layer. For the Homo-Emp. init. method, nodes located deeper than 10.5 mm beneath the outer surface were approximately defined as GM layer. This threshold was determined based on the average thickness of the scalp, skull, and cerebrospinal fluid measured from the MRI images used in this study. For the SOB init. method, the GM layer was directly identified according to the tissue labels provided by the SOB model. The generalized linear model (GLM)^57^ was then applied to the reconstructed GM-node time series of each participant for activation analysis:

$$y_{n}=X\beta_{n}+\varepsilon_{n},$$ (7)

where $y_{n}$ denotes the reconstructed ${\Delta C}_{\mathrm{HbO}}$ time series at GM node n, X denotes the design matrix constructed according to the breath-holding paradigm; $\beta_{n}$ and $\varepsilon_{n}$ denote the estimated regression coefficient vector and residual error, respectively. The task-related β coefficient was extracted for each GM node as the activation feature. For each reconstruction method, one-sample t-tests against zero were performed on the task-related β values across participants at each GM node to assess the statistical significance of task-related activation. GM nodes with p<0.05 were considered significantly activated, and their significance levels were compared between Homo-Emp. init. and SOB init. to evaluate the ability of each method to identify task-evoked hemodynamic responses in the GM layer.

#### 2.5.3 SVM-based task/rest classification

To further evaluate the ability of hemodynamic features obtained from the two reconstruction methods to discriminate between task and rest states, GM-layer features were extracted from the Homo-Emp. init. and SOB init. reconstruction results, including activation β values, kurtosis, and skewness. Within each training fold of the cross-validation, GM nodes were ranked according to the β value for each training sample, and the top 100 GM nodes were selected. The corresponding feature values were then used as inputs to train support vector machine (SVM)^58^ classifiers with a Gaussian radial basis function (RBF) kernel. For the corresponding test fold, the same within-sample node-ranking and feature-extraction rule was applied, and features from the top 100 GM nodes with the highest β values in each test sample were extracted for classification. To ensure a fair comparison, the same feature extraction procedure, cross-validation partitions, and classification pipeline were applied to both reconstruction methods. Classification performance was evaluated using eight-fold cross-validation and quantified by accuracy, area under the receiver operating characteristic (ROC) curve (AUC), F1 score, precision, recall, and specificity.

#### 2.5.4 Dynamic 3D hemodynamic analysis in the GM and SS layers

To further evaluate the spatial localization ability and quantitative sensitivity of the SOB init. method, this study analyzed dynamic 3D ${\Delta C}_{\mathrm{HbO}}$ responses in the GM layer during the *in vivo* breath-holding task. First, the ${\Delta C}_{\mathrm{HbO}}$ responses from the three repeated task blocks were temporally aligned and averaged for each participant to reduce trial-to-trial variability. The averaged responses were then further averaged across all participants to obtain the group-level dynamic ${\Delta C}_{\mathrm{HbO}}$ response. On this basis, the mean ${\Delta C}_{\mathrm{HbO}}$ during the breath-holding task was calculated using a 6s sliding time window. As shown in Fig. 5(f), the 30 s resting-state mean ${\Delta C}_{\mathrm{HbO}}$ was first calculated using the pre-task resting baseline as the reference. The mean ${\Delta C}_{\mathrm{HbO}}$ during the breath-holding task was then calculated using a 6s sliding time window. To characterize the relative changes in the main response region, the top 100 GM nodes with the strongest ${\Delta C}_{\mathrm{HbO}}$ responses within the task time window were selected, and their amplitude changes relative to the first 30 s resting period after the start of each trial were calculated: relative increase = $[(\text{mean}{\text{Δ}C}_{\mathrm{HbO},\text{task}}-\text{mean}\;{\text{Δ}C}_{\mathrm{HbO},\text{rest}})/\text{mean}\;{\text{Δ}C}_{\mathrm{HbO},\text{rest}}]\times 100\%$. This metric was used to characterize the dynamic evolution of the main activated regions. Because the breath-holding task may induce extracerebral and systemic hemodynamic changes through respiratory and autonomic regulation, an additional SS-layer reconstruction analysis was performed to evaluate the potential influence of superficial hemodynamic responses on the GM-layer reconstruction results. Considering the depth-dependent sensitivity of source-detector separations in diffuse optical measurements, 1NN channels, which are primarily sensitive to superficial tissues, were used for SS-layer reconstruction, with the same preprocessing pipeline as that used for the GM-layer analysis. To further evaluate whether superficial physiological fluctuations could affect GM-layer reconstruction, a controlled simulation is provided in Section S6 of the Supplementary Material to assess the effects of SS-layer interference on GM-layer reconstruction.

#### 2.5.5 Quantitative reconstruction metrics

For the numerical simulation and phantom experiments, reconstruction quality was quantitatively evaluated using mean absolute error (MAE), structural similarity index (SSIM), and contrast-to-noise ratio (CNR). MAE quantifies node-wise reconstruction error, SSIM assesses structural fidelity, and CNR evaluates target-to-background contrast relative to regional fluctuations:

$$\left\{ \begin{array}{l} \mathrm{MAE}=\overset{N^{\left(S\right)}}{\underset{n=1}{\sum }}|\Delta \mu_{\text{real}\left(n\right)}-\Delta \mu_{\mathrm{recon}\left(n\right)}|/N^{\left(S\right)}\\ \mathrm{SSIM}=\frac{\left(2{\overset{¯}{\Delta \mu }}_{\text{real}}{\overset{¯}{\Delta \mu }}_{\mathrm{recon}}+c_{1})(2\sigma_{\text{real},\mathrm{recon}}+c_{2}\right)}{({{\overset{¯}{\Delta \mu }}^{2}}_{\text{real}}+{{\overset{¯}{\Delta \mu }}^{2}}_{\mathrm{recon}}+c_{1})\left({\sigma^{2}}_{\text{real}}+{\sigma^{2}}_{\mathrm{recon}}+c_{2}\right)},\\ \mathrm{CNR}=\frac{\left|{\overset{¯}{\Delta \mu }}_{\mathrm{ROI}}-{\overset{¯}{\Delta \mu }}_{\mathrm{ROB}}\right|}{\sqrt{\omega {\sigma^{2}}_{\mathrm{ROI}}+\left(1-\omega \right){\sigma^{2}}_{\mathrm{ROB}}}},\;\omega =V_{\mathrm{ROI}}/(V_{\mathrm{ROI}}+V_{\mathrm{ROB}}) \end{array}\right.$$ (8)

where $\Delta \mu_{\text{real}(n)}$ and $\Delta \mu_{\mathrm{recon}(n)}$ denote the true and reconstructed absorption-change distributions at node n, respectively; ${\overset{¯}{\Delta \mu }}_{\text{real}}$ and ${\overset{¯}{\Delta \mu }}_{\text{recon}}$ denote the true and reconstructed absorption-change distributions, respectively,; $N^{\left(S\right)}=16,278$ is the total number of finite-element nodes in the SOB; ${\sigma^{2}}_{\text{real}}$ and ${\sigma^{2}}_{\mathrm{recon}}$ represent the variances of the true and reconstructed absorption-change distributions, respectively,; $\sigma_{\text{real},\mathrm{recon}}$ is their covariance; $c_{1}$ and $c_{2}$ are stabilizing constants introduced to avoid division by zero; ${\overset{¯}{\Delta \mu }}_{\mathrm{ROI}}$ and ${\overset{¯}{\Delta \mu }}_{\mathrm{ROB}}$ denote the mean reconstructed absorption changes within the target and background regions, respectively,; and $V_{\mathrm{ROI}}$ and $V_{\mathrm{ROB}}$ denote the volumes of the target and background regions, respectively. A lower MAE, an SSIM closer to 1, and a higher CNR indicate better reconstruction performance.

## 3 Results

### 3.1 Performance Evaluation of the SOB Model

#### 3.1.1 Anatomical fidelity

**Fig. 6:**
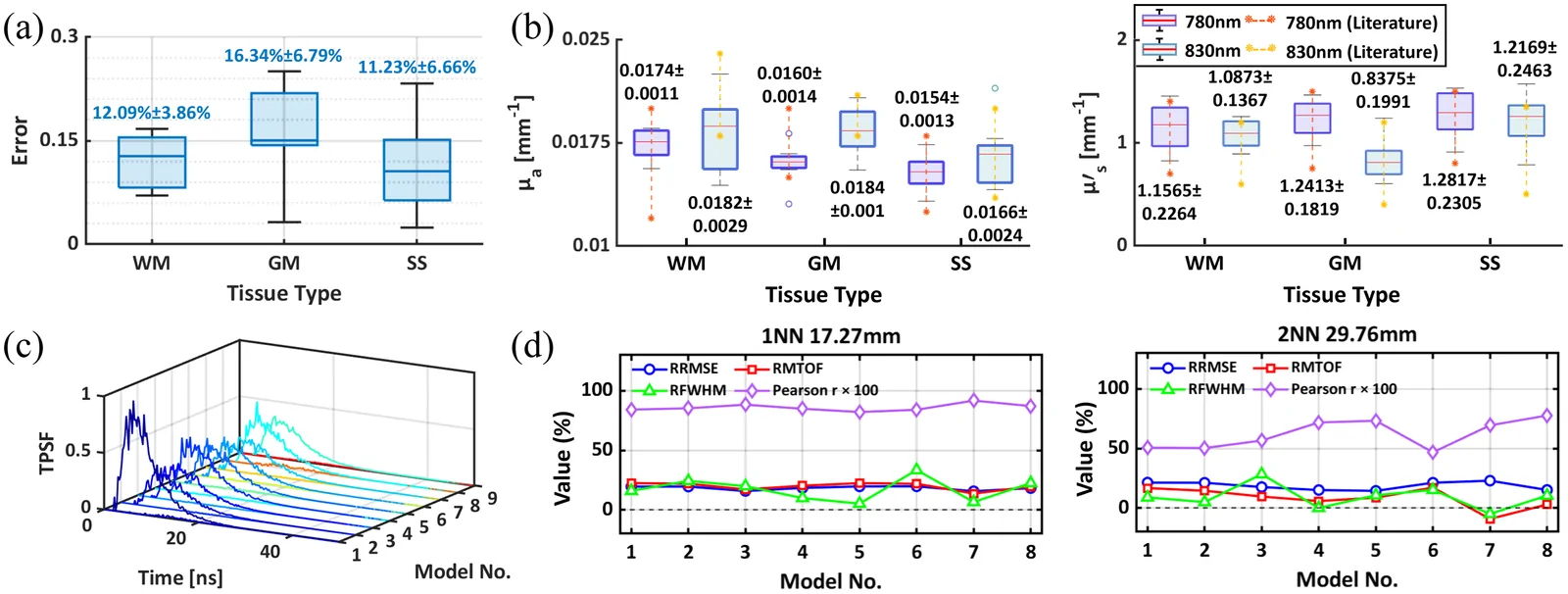
Performance evaluation of the standard optical brain model. (a) Anatomical fidelity, quantified by the proportion of non-overlapping nodes in the SS, GM, and WM layers. (b) Comparison of TD-DOT reconstructed tissue optical properties with literature-reported values for SS, GM, and WM.^35^^–^^41^ (c) Comparison of simulated temporal point-spread functions curves among different models; (d) Quantitative comparison of TPSF consistency between the standardized and individual digital brain models for 1NN and 2NN source-detector separations, evaluated using relative root mean square error (RRMSE), relative mean time-of-flight error (RMTOF), relative full width at half maximum error (RFWHM), and Pearson correlation coefficient r. Pearson correlation values are multiplied by 100 for visualization.

As shown in Fig. 6(a), the mean proportion of nonoverlapping nodes between the standardized and original DB models was below 16.5% across all tissues.

#### 3.1.2 Tissue-specific optical property consistency

As shown in Fig. 6(b), the reconstructed tissue optical properties were compared with literature-reported^35^^–^^41^ ranges for superficial head tissues, GM, and WM. The SS-layer reference values were mainly based on reported optical properties of superficial human head tissues,^36^ whereas the GM and WM reference values were derived from published human brain optical-property studies.^39^ Overall, the reconstructed $\mu_{a}$ and $\mu_{s}^{\prime }$ were within or close to the literature-reported ranges across different tissue layers. A slight deviation was observed in the $\mu_{s}^{\prime }$ of WM.

#### 3.1.3 Photon-transport consistency

Figure 6(c) shows that the TPSF curves simulated from the standard DB model had slightly higher amplitudes than those from the individual DB models, but their overall profiles were consistent. As shown in Fig. 6(d), quantitative TPSF evaluation showed that, for the 1NN channels, the RRMSE, RMTOF, and RFWHM were 18.33%±1.70%, 19.91%±3.10%, and 17.22%±9.71%, respectively, with a Pearson r of 0.86±0.03. For the 2NN channels, the RRMSE was 18.59%±3.44%, similar to that of the 1NN channels, whereas the RMTOF and RFWHM were 8.26%±8.70% and 9.19%±10.08%, respectively. The Pearson r decreased to 0.62±0.12 for the 2NN channels. These results indicate that the standard DB model generally preserved the main photon-transport characteristics of the individual DB models, particularly in terms of temporal shift and temporal broadening metrics.

### 3.2 Numerical Simulation

**Fig. 7:**
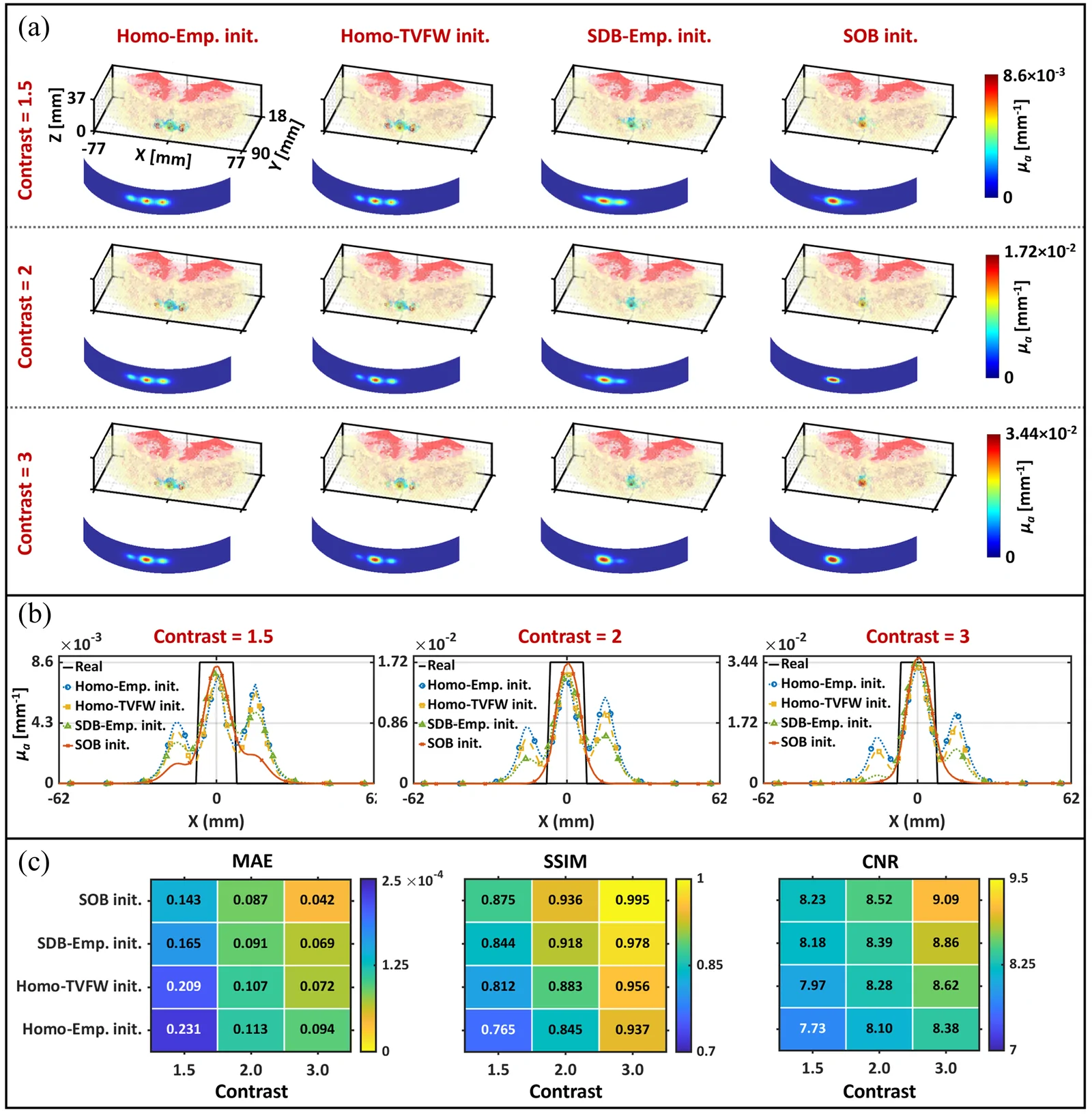
Reconstruction performance of different initialization strategies in the single-target simulation under varying absorption contrast ratios. Four initialization strategies were compared at target-to-background absorption contrasts of 1.5, 2.0, and 3.0: homogeneous-background empirical initialization (Homo-Emp. init.), homogeneous-background tissue-volume-fraction-weighted initialization (Homo-TVFW init.), standard-digital-brain empirical initialization (SDB-Emp. init.), and standard-optical-brain initialization (SOB init.). (a) Reconstructed 3D and 2D absorption-change images. (b)Corresponding cross-sectional profiles compared with the ground truth. (c)Quantitative comparison of MAE, SSIM, and CNR.

The performance of four reconstruction methods, namely Homo-Emp. init., Homo-TVFW init., SDB-Emp. init., and SOB init., was compared under three absorption contrast ratios. The 3D and two-dimensional (2D) reconstruction maps and corresponding profile curves are shown in Figs. 7(a) and 7(b). Across all contrast conditions, Homo-TVFW init. showed only a slight improvement in amplitude recovery compared with Homo-Emp. init.; however, its reconstruction results still exhibited spatial spreading, artifacts, and localization errors. The corresponding profile curves also showed evident broadening and peak-position shifts, indicating limited improvement over the homogeneous-background reconstruction. In contrast, SDB-Emp. init. yielded improved target localization and spatial focusing, even when empirical initial optical parameters were used. Both the reconstructed target region and the profile peak were closer to the true target location. SOB init. incorporated tissue-specific optical-property initialization into the SDB anatomical background, resulting in a more compact reconstructed target, clearer target boundaries, and profile curves that showed the closest agreement with the true distribution.

The quantitative metrics shown in Fig. 7(c), including MAE, SSIM, and CNR, showed a consistent trend across the three absorption contrast ratios. Overall, the reconstruction performance followed the order of SOB init. > SDB-Emp. init. > Homo-TVFW init. > Homo-Emp. init. Compared with Homo-Emp. init., Homo-TVFW init. provided only limited improvement, whereas SDB-Emp. init. outperformed both homogeneous-background methods, suggesting that incorporation of the standard DB anatomical background improved reconstruction stability and localization accuracy. Among all methods, SOB init. achieved the best performance under all three absorption contrast conditions. As the absorption contrast increased from 1.5 to 3.0, the MAE obtained using SOB init. decreased from 0.143 to 0.042, while SSIM increased from 0.875 to 0.995 and CNR increased from 8.23 to 9.09. Compared with Homo-Emp. init., SOB init. reduced MAE by approximately 38.1%, 23.0%, and 55.3% at absorption contrast ratios of 1.5, 2.0, and 3.0, respectively. These results demonstrate that SOB init. improved reconstruction accuracy, structural fidelity, and contrast recovery in the simulation experiments.

### 3.3 Phantom Validation under Real Measurement Conditions

**Fig. 8:**
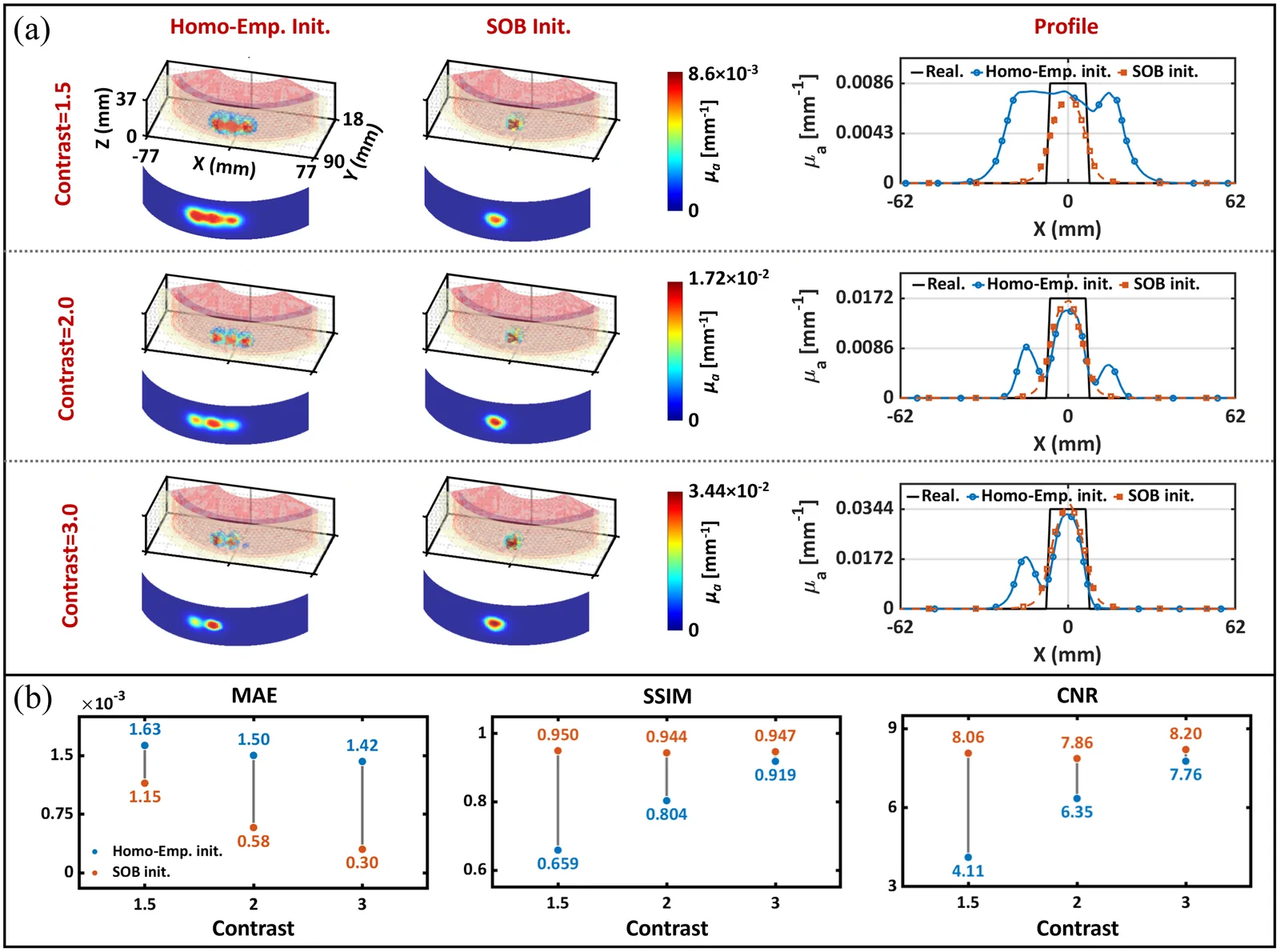
Single-target phantom reconstruction under three absorption-contrast conditions. Homogeneous-background empirical initialization (Homo-Emp. init.) and standard-optical-brain initialization (SOB init.) were compared at target-to-background absorption contrasts of 1.5, 2.0, and 3.0. (a) Reconstructed 3D and 2D absorption-change images and corresponding cross-sectional profiles. (b) Quantitative comparison of MAE, SSIM, and CNR.

The phantom reconstruction results are shown in Fig. 8(a). Homo-Emp. init. produced pronounced artifacts and shape distortion, particularly under low-contrast conditions. At a contrast ratio of 1.5, the reconstructed region obtained using Homo-Emp. init. was markedly diffused, and the cross-sectional profile exhibited a broad plateau-like distribution with a substantial deviation from the true target boundary. As the contrast ratio increased to 2.0 and 3.0, the main peak location obtained using Homo-Emp. init. improved, but lateral false peaks and boundary broadening were still observed. In contrast, SOB init. produced a more concentrated reconstructed distribution across all three contrast conditions, with better preservation of target morphology and clearer target boundaries. The cross-sectional profiles further showed that the peak location, curve width, and reconstructed target contour obtained using SOB init. were more consistent with the true target distribution.

The quantitative metrics are shown in Fig. 8(b). Across all contrast levels, SOB init. achieved consistently lower MAE, higher SSIM values closer to 1, and higher CNR than Homo-Emp. init. Specifically, at contrast ratios of 1.5, 2.0, and 3.0, the MAE values obtained using Homo-Emp. init. were 1.63, 1.50, and 1.42, respectively, whereas those obtained using SOB init. decreased to 1.15, 0.58, and 0.30. Correspondingly, the SSIM values increased from 0.659, 0.804, and 0.919 for Homo-Emp. init. to 0.950, 0.944, and 0.947 for SOB init., respectively. The CNR values also increased from 4.11, 6.35, and 7.76 to 8.06, 7.86, and 8.20, respectively. These results indicate that SOB init. improved target localization, morphological recovery, boundary preservation, and quantitative reconstruction performance under experimental conditions.

### 3.4 *In Vivo* Validation of SOB-Guided Distance-Dependent CW-fDOT

#### 3.4.1 GM-layer activation sensitivity

**Fig. 9:**
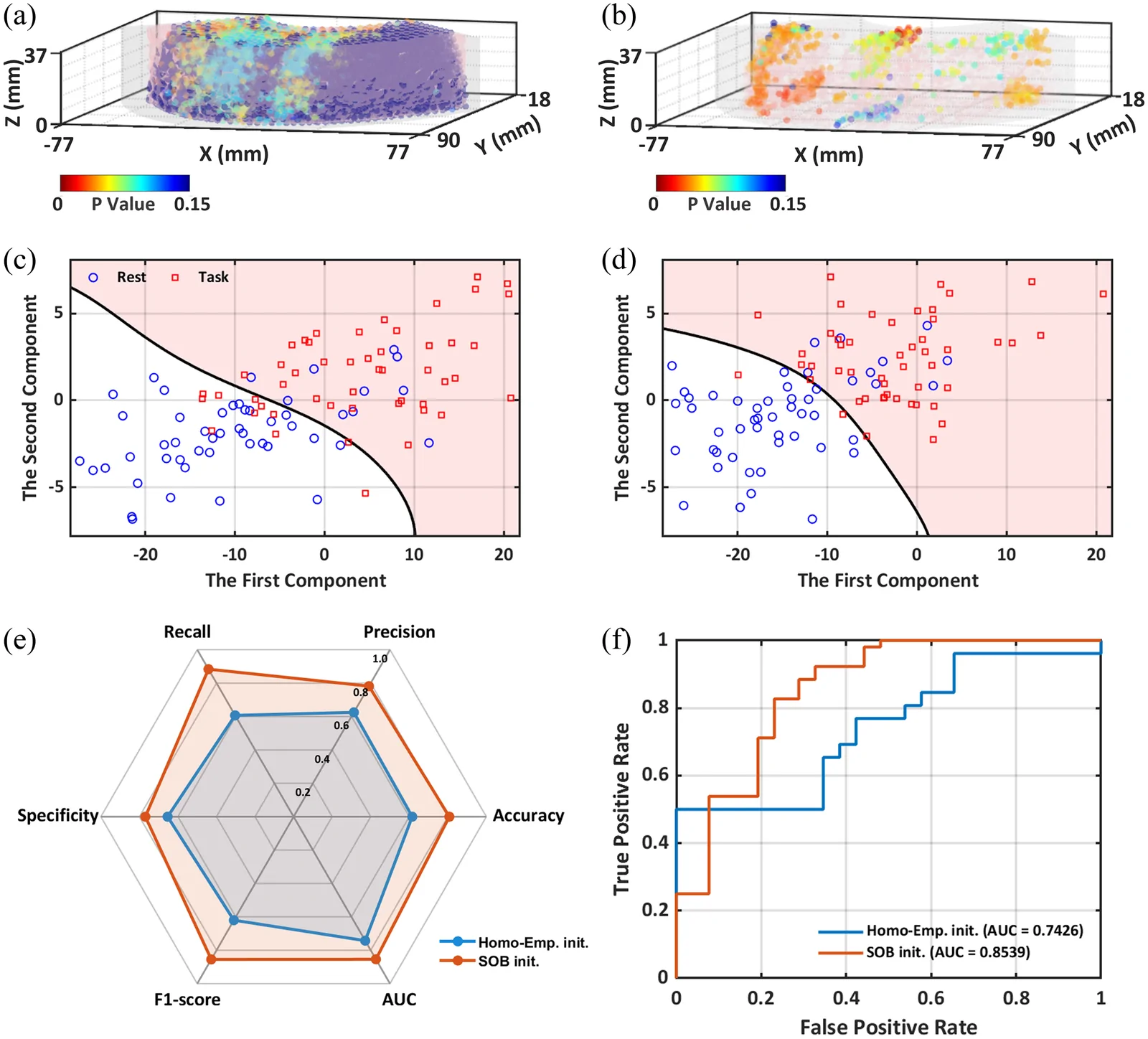
*In vivo* comparison of GM-layer activation and task/rest classification performance. (a,b) GM-layer task-related activation maps based on one-sample t-tests against zero for β values obtained using (a) Homo-Emp. init. and (b) SOB init. (c,d) PCA-projected task/rest feature distributions and SVM decision boundaries obtained using (c) Homo-Emp. init. and (d) SOB init. (e) Comparison of classification metrics between the two reconstruction methods, including accuracy, AUC, F1 score, precision, recall, and specificity. (f) ROC curves for Homo-Emp. init. and SOB init.

As shown in Figs. 9(a) and 9(b), compared with Homo-Emp. init., SOB init. generally yielded lower p-values in GM nodes and increased the number of significantly activated GM nodes by 79.52%. This result indicates that SOB init. improved the sensitivity for detecting task-related GM-layer hemodynamic activation.

#### 3.4.2 Task/rest classification performance

As shown in Figs. 9(c) and 9(d), in the 2D principal component analysis (PCA)-projected feature space, the features obtained using Homo-Emp. init. were relatively dispersed, with substantial overlap between the rest and task states. In contrast, SOB init. produced more compact and better-separated feature clusters, together with a clearer SVM decision boundary. The quantitative results in Fig. 9(e) showed that SOB init. achieved values above 0.8 across all classification metrics, including accuracy, AUC, F1 score, precision, recall, and specificity, whereas the corresponding values for Homo-Emp. init. ranged from 0.6 to 0.75. The ROC curves in Fig. 9(f) further showed that SOB init. yielded a curve generally higher than that of Homo-Emp. init., indicating that the GM-layer hemodynamic features extracted using SOB init. had better discriminative ability for task/rest classification.

#### 3.4.3 Dynamic 3D distributions of hemodynamic concentration changes

**Fig. 10:**
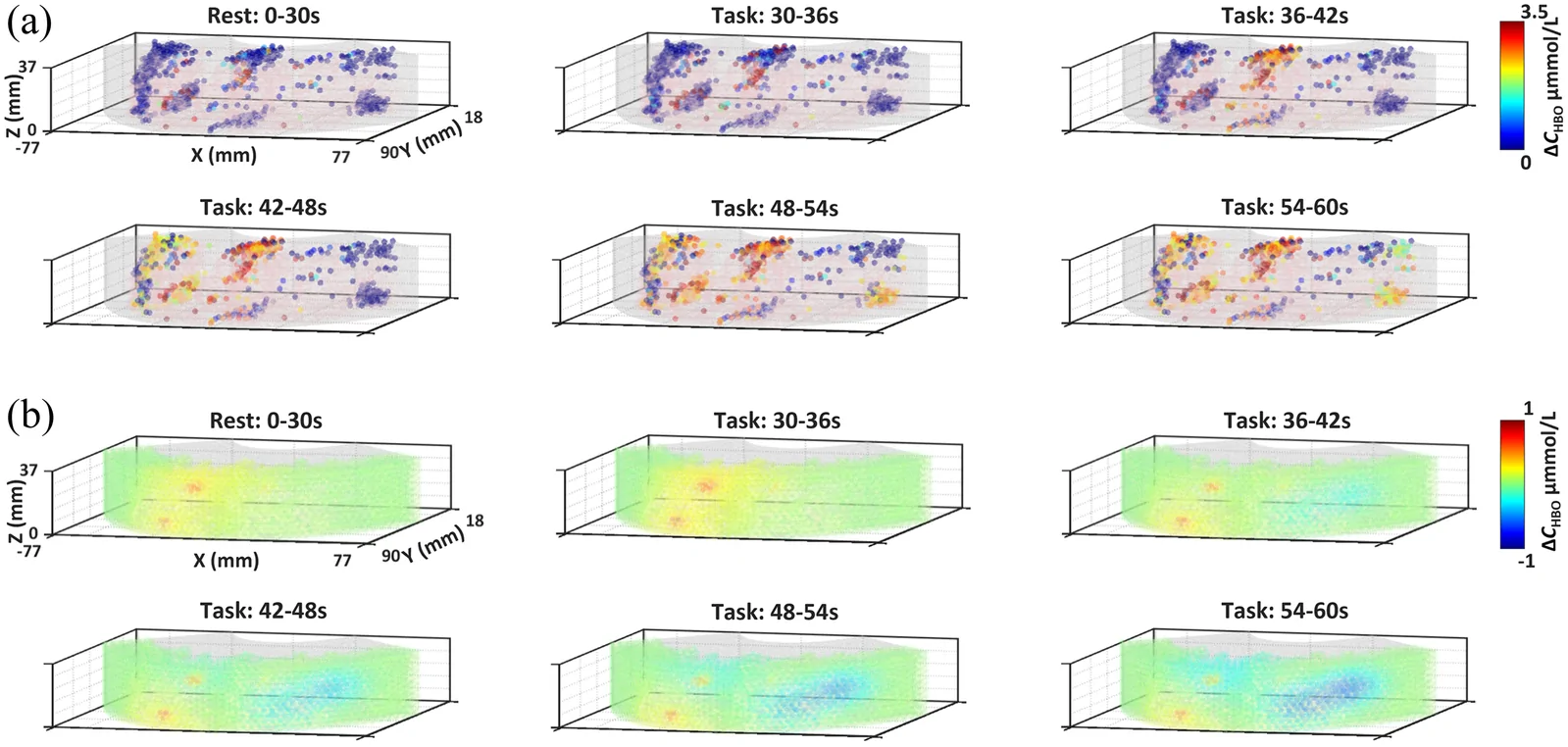
Dynamic 3D distributions of ${\Delta C}_{\mathrm{HbO}}$ during the breath-holding task reconstructed using the SOB init. The 30s resting period within each trial was used as the baseline reference. The 0 to 30 s interval denotes the resting baseline within each trial, whereas the 30 to 60s breath-holding period was divided into five consecutive nonoverlapping 6s windows: 30 to 36, 36 to 42, 42 to 48, 48 to 54, and 54 to 60s. (a)GM-layer. (b) SS-layer.

As shown in Fig. 10(a), the GM-layer ${\Delta C}_{\mathrm{HbO}}$ response increased by 15.42% across six consecutive time windows, with 98.47% of this increase occurring within the first two windows after task onset (30 to 36 s and 36 to 42 s). The main response was concentrated in the superior portion of the right middle frontal region. The SS-layer reconstruction results are shown in Fig. 10(b). During the resting period and the early phase of breath-holding, only 4.93% increase in ${\Delta C}_{\mathrm{HbO}}$ was observed in the right superficial region. At 36 to 42 s after task onset, a decrease in ${\Delta C}_{\mathrm{HbO}}$ appeared in the left superficial region and gradually expanded and deepened as breath-holding continued. Compared with the GM-layer response, the SS-layer ${\Delta C}_{\mathrm{HbO}}$ changes were more spatially diffuse and smaller in magnitude.

## 4 Discussion

This study developed and validated a SOB-guided distance-dependent CW-fDOT framework to improve reconstruction accuracy, functional localization, and hemodynamic sensitivity without requiring subject-specific anatomical or optical-property data. Model evaluation confirmed the feasibility of the SOB model as a reusable prior, while numerical simulations, phantom experiments, and *in vivo* breath-holding validation demonstrated that incorporating the SOB prior consistently enhanced CW-fDOT reconstruction performance.

### 4.1 Feasibility of the SOB Model as a Prior

The results showed that the SOB model could effectively preserve the major anatomical structures, tissue optical properties, and photon transport characteristics required for CW-fDOT reconstruction. Anatomical fidelity analysis showed that the proportion of nonoverlapping nodes between the standardized DB model and the individual DB models was below 16.5% across all tissue. This indicates that, although the standardized model cannot fully reproduce fine inter-subject anatomical variations, it can preserve the major spatial distribution of frontal tissue and provide a relatively stable anatomical prior for CW-fDOT reconstruction. In terms of tissue optical properties, the $\mu_{a}$ and $\mu_{s}^{\prime }$ reconstructed by TD-DOT were generally close to the ranges reported in the literature,^35^^–^^41^ indicating that the estimated properties were physiologically reasonable. A slight deviation was observed in the $\mu_{s}^{\prime }$ of white matter, which may be related to differences in measurement techniques, sample conditions, and inter-subject variability. This also suggests that properties may be more susceptible to methodological factors and should be further validated using larger cohorts and broader wavelength ranges.

The comparison of MC-generated TPSF curves between the standard DB model and the individual DB models further supports the feasibility of using the SOB model as a prior for CW-fDOT. The two sets of simulated TPSFs showed good overall consistency, indicating that the standard DB can preserve the main temporal characteristics of photon propagation. Quantitative comparisons showed that, at shorter source-detector separations, the TPSF differences were relatively small, with lower RRMSE, RMTOF, and RFWHM values and higher Pearson correlation coefficients. These findings indicate that photon transport characteristics in superficial and intermediate-depth regions were well preserved. At longer separations, the overall pointwise deviations remained limited, and the MTOF and temporal broadening of photon propagation were also well maintained. However, the Pearson correlation coefficients decreased, possibly because long-separation channels are more sensitive to deeper tissues and involve longer photon paths, leading to the accumulation of individual anatomical differences along the propagation pathways.

Overall, the SOB model preserved the key anatomical, optical, and photon transport characteristics required for CW-fDOT reconstruction. It can therefore serve as a physiologically reasonable prior model for improving reconstruction stability and accuracy. To support future reuse, the SOB model is publicly available (DOI: 10.5281/zenodo.20825735). Meanwhile, the results also indicate that the SOB cannot completely eliminate subject-specific anatomical differences, particularly in deeper tissues or along longer photon propagation paths. Future studies should expand the sample size, develop more refined anatomical modeling strategies, and optimize tissue optical-property estimation to improve the generalizability of the SOB model.

### 4.2 Mechanistic Contributions of SOB Anatomical and Optical Priors

The comparison of the four initialization strategies helps clarify the relative contributions of the anatomical background model and the initial optical properties to CW-fDOT reconstruction. Although Homo-TVFW init. used initial optical properties that were closer to the true tissue-averaged values under a homogeneous background model, its improvement over Homo-Emp. init. was limited. The reconstructed results still showed evident spatial spreading, artifacts, and localization bias. This indicates that, under the homogeneous background assumption, optimizing only the initial optical properties is insufficient to eliminate the photon-transport model mismatch caused by the simplified anatomical model.

In contrast, SDB-Emp. init. substantially improved target localization and spatial focusing even when empirical initial optical properties were used, with both the reconstructed region and profile peak located closer to the true target center. This suggests that incorporating the SDB anatomical background model provides spatial constraints that better reflect the actual photon propagation environment in the head, thereby suppressing the spread of absorption changes into physiologically implausible regions and improving structural accuracy and spatial fidelity. Therefore, compared with optimizing the initial optical properties alone, the anatomical background model plays a more critical role in improving CW-fDOT reconstruction performance. On the basis of the SDB anatomical background, SOB init. further incorporated tissue-specific optical-property initialization, resulting in improved spatial compactness, clearer target boundaries, and more stable amplitude recovery. This indicates that the anatomical structural prior mainly improves the spatial plausibility of the photon sensitivity distribution, whereas the tissue-specific optical properties further enhance the accuracy of the forward model in simulating the actual photon propagation process. The combination of these two priors can reduce errors jointly caused by geometric and optical-property deviations, thereby improving the quantitative accuracy and robustness of inverse reconstruction.

In addition, real brain functional activity often involves multiple spatially separated cortical regions rather than a single focal activation. To evaluate the applicability of the proposed framework to complex activation patterns, we performed dual-target simulations. The results (Supplementary Material, Fig. S3) showed that SOB init. maintained good localization accuracy and morphological fidelity under different absorption contrasts, indicating that the proposed framework has the potential to enhance the reliability of reconstructing complex cortical hemodynamic patterns.

### 4.3 Functional Sensitivity, Classification Performance and 3D Dynamic Hemodynamics

This study adopted a progressive validation strategy from numerical simulations to physical phantom experiments and finally to *in vivo* breath-holding experiments. Numerical simulations provided controlled conditions with known ground truth, enabling direct quantitative comparisons among different initialization strategies. Phantom experiments introduced real source-detector coupling variability and system noise while preserving known target geometry and absorption contrast, thereby evaluating the performance of the proposed method under realistic experimental conditions. *In vivo* experiments further examined whether the improved reconstruction performance could be translated into functional hemodynamic detection and brain-state discrimination.

Across these validation experiments, SOB init. consistently outperformed the conventional Homo-Emp. init. method. In numerical simulations and phantom experiments, the improvement was mainly reflected in more accurate target localization, clearer boundaries, better profile agreement, higher structural fidelity, and stronger contrast recovery. These results indicate that the SOB prior improves reconstruction quality not only under idealized simulation conditions but also under realistic measurement conditions involving system noise and experimental variability. The *in vivo* breath-holding experiment further demonstrated the functional relevance of this reconstruction improvement. Compared with Homo-Emp. init., SOB init. improved the identification of activated regions. In task/rest classification, SOB init. produced more compact and better separated GM-layer features in the PCA-projected feature space, and improved SVM classification metrics, including accuracy, AUC, F1 score, precision, recall, and specificity. These findings suggest that introducing SOB can enhance functional-state classification, which is important for future brain-state monitoring, neurocognitive assessment, and portable neuroimaging workflows. The 3D dynamic GM-layer ${\Delta C}_{\mathrm{HbO}}$ results during the breath-holding task showed that ${\Delta C}_{\mathrm{HbO}}$ increased by 15.42% after task onset, with most of the response enhancement occurring in the early time window. The main reconstructed response was located in the superior region of the right middle frontal gyrus, consistent with the involvement of the prefrontal region in autonomic regulation induced by respiratory control. This indicates that the SOB-guided CW-fDOT framework can track the 3D spatial evolution of task-evoked hemodynamic responses in the GM layer.

In addition, because the breath-holding task may induce systemic physiological responses, including respiratory regulation, changes in arterial ${\mathrm{CO}}_{2}$, and alterations in superficial vascular oxygenation, we further analyzed SS-layer ${\Delta C}_{\mathrm{HbO}}$ responses based on the 1NN data. The results showed that, compared with the GM layer, SS-layer ${\Delta C}_{\mathrm{HbO}}$ changes were generally more diffuse, had smaller amplitudes, and exhibited different spatial distribution patterns. These findings indicate that superficial hemodynamic responses during breath holding differ from the main GM-layer activation response in both spatial morphology and response intensity, suggesting that superficial hemodynamic changes contributed relatively little to the GM-layer reconstruction results. However, the *in vivo* SS-layer analysis alone cannot completely exclude the potential influence of superficial physiological interference on GM-layer reconstruction. Therefore, this study further evaluated the effect of SS-layer interference on GM-layer reconstruction under controlled simulation conditions. The results (Supplementary Material, Fig. S4) showed that, after introducing SS-layer physiological interference, the reconstructed GM-layer activation region remained close to the true target location, with well-preserved spatial morphology and no obvious localization shift. Quantitative evaluation also showed that SS-layer interference caused only limited degradation in reconstruction quality. These results indicate that the proposed SOB-guided distance-dependent CW-fDOT reconstruction framework can maintain stable GM-layer localization and contrast recovery in the presence of superficial physiological interference, demonstrating a certain degree of robustness against SS-layer interference. This robustness may be related to the combined effects of the distance-weighting strategy and the tissue-structural priors provided by the SOB model. The distance-weighting strategy can make use of the depth-dependent sensitivity of different source–detector separations, while the SOB model provides anatomical constraints for the SS, GM, and WM layers. Together, these factors help distinguish superficial and deep signals more appropriately during reconstruction, thereby reducing the influence of SS-layer interference on GM-layer localization. Nevertheless, superficial physiological interference remains an important issue in fNIRS and CW-fDOT studies.^59^ Future work should incorporate denser channel coverage, short-separation regression, and simultaneous physiological monitoring to further improve the separation of cortical activation from superficial and systemic physiological responses.

### 4.4 Practical Implications, Limitations and Future Work

The main value of the proposed framework is that it improves CW-fDOT reconstruction performance while preserving the portability, low cost, and ease of use of CW-fNIRS systems. Although subject-specific anatomy and optical parameters can improve reconstruction accuracy, they increase experimental cost, prolong data acquisition, and complicate the workflow, limiting their use in large-scale studies, repeated measurements, and clinical screening. In contrast, the SOB-guided framework incorporates anatomical and optical priors into CW-fDOT by scaling the SOB according to individual head dimensions. This approach achieves a practical balance among anatomical realism, reliable parameter initialization, portability, and feasibility. Therefore, it may facilitate broader applications of CW-fDOT in cognitive neuroscience, neurocognitive assessment, brain-state monitoring, and clinical screening.

Several limitations should be acknowledged, and future work may further advance this framework in the following aspects. First, the current SOB model was constructed from data acquired from only eight healthy volunteers. It should therefore be regarded as a proof-of-concept population-standardized model and methodological validation framework, rather than a final optical brain atlas with sufficient population representativeness. Future studies should establish a dedicated multimodal database for SOB modeling, including participants with diverse sex, age, geographic background, and head-size characteristics. In addition to high-resolution structural MRI data, head-surface dimensional parameters and spatial registration information matched to the TD-DOT/CW-fDOT measurement regions should be systematically collected. Sample-size determination should not rely solely on a fixed threshold but should comprehensively consider the stability of anatomical statistics, tissue optical properties, TPSF characteristics, and CW-fDOT reconstruction performance as the sample size increases. Open MRI datasets from healthy populations that preserve the complete outer head contour and provide sufficient spatial resolution may serve as useful supplements. Disease-related datasets, such as brain tumor segmentation challenge databases,^60^ may also be used to explore the effects of pathological brain structures on model registration, photon-transport simulation, and functional reconstruction accuracy.

Second, this study mainly evaluated the contribution of SOB priors within an MC-based photon-transport framework. Whether SOB priors can produce consistent improvements under different photon-transport models,^61^ background structural assumptions, and optical-property settings remains to be systematically investigated. Future studies should also optimize SOB-based individualized registration and correction methods. The current framework primarily scales the SOB model according to a limited number of individual head-size parameters to obtain approximate structural priors. In future work, additional head-surface anatomical landmarks, optode position information, and individual head-shape parameters may be incorporated to improve the spatial correspondence between the SOB model and the actual head anatomy. Signal correction methods, such as short-channel regression, may also be introduced to reduce extracerebral interference, including scalp-related signals. These strategies are expected to improve robustness, individual adaptability, and the reproducibility of inter-subject comparisons under real CW-fDOT measurement conditions without substantially increasing experimental complexity.

Third, the current CW-fDOT method measures changes in hemoglobin concentration rather than absolute concentrations. The amplitude of the reconstructed signals may be affected by individual anatomical differences, variations in tissue optical properties, signal drift, and other factors. Therefore, although SOB priors improved reconstruction stability and spatial localization, comparisons of absolute signal amplitudes should still be interpreted with caution. Finally, the current *in vivo* validation was mainly based on a frontal breath-holding paradigm. This paradigm is simple, reproducible, and capable of inducing relatively stable hemodynamic responses; however, its response pattern cannot fully represent cortical activation during complex cognitive, sensory, or motor tasks. Therefore, the present results primarily support the feasibility and effectiveness of the SOB-guided framework for reconstructing frontal hemodynamic responses. Future studies should extend the frontal SOB model to multiple brain regions and ultimately to the whole brain, while incorporating more complex cognitive, sensory, motor, and clinical task paradigms. This will help systematically evaluate the stability and applicability of SOB priors over larger modeling domains, different source–detector configurations, and more complex light-propagation conditions, thereby promoting the transition of this framework from local methodological validation to brain-network studies and clinical functional assessment.

## 5 Conclusion

This study proposed an SOB-guided quantitative CW-fDOT framework that integrates MRI-derived frontal anatomical statistics and TD-DOT-measured tissue optical properties as reusable anatomical and optical priors for CW-fDOT reconstruction. By reducing the dependence on subject-specific anatomical structures and optical-property measurements, this framework improves reconstruction accuracy and functional information extraction while preserving the portability, low cost, and operational simplicity of CW systems. This approach provides a feasible pathway toward more precise quantitative functional brain imaging and may support future applications in cognitive neuroscience, brain-state monitoring, and clinical screening.

## Supplementary Material

10.1117/1.NPh.13.3.035011.s01

## Data Availability

The standard optical brain model and related code have been made publicly available in the public data repository Zenodo, with the DOI: 10.5281/zenodo.20825735.
