# Supplementary material for: CW-fDOT with a standard optical brain model: improving hemodynamic sensitivity and functional localization
Source: Neurophotonics. 2026 Sep 22;13(3):035011. doi: 10.1117/1.NPh.13.3.035011 (PMC13597020; doi:10.1117/1.NPh.13.3.035011)
Supplement: Supplementary file 1 [file NPh_013_035011_SD001.pdf]

Supplementary Material

This supplementary material provides additional methodological details and validation results supporting the SOB init. framework described in the main text. It includes the region-based TD-DOT reconstruction algorithm for estimating SS, GM, and WM optical properties; the TD-DOT system configuration and adaptive acquisition strategy; MC simulation settings for TPSF analysis and CW-fDOT reconstruction; and additional simulations evaluating dual-target reconstruction performance and robustness against SS-layer physiological interference. These materials provide technical support for the anatomical and optical priors used in SOB-guided CW-fDOT reconstruction and further demonstrate the spatial localization ability and robustness of the proposed framework.

S1 Region-based TD-DOT

The purpose of TD-DOT reconstruction was to estimate the optical properties of the SS, GM, and WM regions, which were subsequently used as optical priors for CW-fDOT reconstruction within the SOB init. framework. A generalized pulse spectrum technique (GPST)-based method was used, in which TD signals were first transformed into the Laplace domain and then inverted in the complex frequency domain. Two real transformation factors, p_1 and p_2, were introduced to improve computational efficiency and separate absorption and scattering effects.[42]

S1.1 Data calibration

A size-matched reference phantom was measured using the same acquisition protocol as the target to reduce system-related effects, including the instrument response function (IRF) and source-detector coupling variations. The calibrated transformed data were calculated as follows:

$$\hat{\Gamma}(\xi_d,\zeta_s,p_i)=\frac{\hat{\Gamma}_{\text{Tar}}(\xi_d,\zeta_s,p_i)}{\hat{\Gamma}_{\text{Ref}}(\xi_d,\zeta_s,p_i)}\times\Gamma_{\text{M}}(\xi_d,\zeta_s,p_i)=\frac{\Gamma_{\text{Tar}}(\xi_d,\zeta_s,p_i)\times\text{I}(\xi_d,\zeta_s,p_i)}{\Gamma_{\text{Ref}}(\xi_d,\zeta_s,p_i)\times\text{I}(\xi_d,\zeta_s,p_i)},\ i=1,2, \tag{S1}$$

where $\hat{\Gamma}_{\text{Tar}}(\xi_d,\zeta_s,p_i)$ and $\hat{\Gamma}_{\text{Ref}}(\xi_d,\zeta_s,p_i)$ are the Laplace-transformed data of the target and reference measurements, respectively, and $\Gamma_{\text{Tar}}(\xi_d,\zeta_s,p_i)$ and $\Gamma_{\text{Ref}}(\xi_d,\zeta_s,p_i)$ denote the corresponding IRF-corrected data. $\Gamma_{\text{M}}(\xi_d,\zeta_s,p_i)$ is the model-calculated transformed data for the reference phantom based on its geometry and known optical properties. $\text{I}(\xi_d,\zeta_s,p_i)$ is the IRFs of all source-detector pairs; ξ_d (d=1, …, D) and ζ_s (s=1, …, S) denote the detector and source positions, respectively.

S1.2 GPST-based optical inversion

A modified GPST-based inversion framework was used to recover the optical properties. The discretized Laplace-domain diffusion equation was formulated using an intermediate variable $O(\mathbf{r}_n,p_i)$:[43]

$$\delta\mathbf{\Gamma}(p_i)=\mathbf{J}(p_i)\mathbf{O}(p_i), \tag{S2}$$

where $\delta\mathbf{\Gamma}(p_i)=[(\xi_1,\zeta_2,p_i),(\xi_2,\zeta_1,p_i),...,(\xi_D,\zeta_S,p_i)]$ contains the perturbed data for all source-detector pairs, with $\delta\Gamma(\xi_d,\zeta_s,p_i)=\hat{\Gamma}(\xi_d,\zeta_s,p_i)-\Gamma_{\text{M}}(\xi_d,\zeta_s,p_i)$; $\mathbf{O}(p_i)=[O(\mathbf{r}_1,p_i),O(\mathbf{r}_2,p_i),...,O(\mathbf{r}_N,p_i)]^T$ lists the intermediate variable at all the discrete nodes, and $\mathbf{J}(p_i)$ is the Jacobian matrix with its entry calculated as:

$$J(\xi_d,\zeta_s,\mathbf{r}_n,p)=-\alpha\sum_{\mathbf{r}_n\in\Omega_e}\left\langle\Gamma(\xi_d,\ \mathbf{r}_n,\ p_i)\Phi(\mathbf{r}_n,\ \zeta_s,\ p_i)\right\rangle V(\Omega_e) \tag{S3}$$

where $\Gamma[\xi_d,\mathbf{r}_n,p_i]$ and $\Phi(\mathbf{r}_n,\ \zeta_s,\ p_i)$ are the Green's function for the boundary flux and internal fluence distribution , respectively, calculated using the current optical properties. $V(\Omega_e)$ is the

volume of the e-th element Ω_e containing node $\mathbf{r}_n$; α = 1/4 for the tetrahedral element; $\langle\cdot\rangle$ denotes the mean within Ω_e.

Using the intermediate variables $O(\mathbf{r}_n, p_1)$ and $O(\mathbf{r}_n, p_2)$, the absorption and reduced scattering perturbations were separated as follows: [42]

$$\begin{cases} \delta\mu_a(\mathbf{r}_n) = \dfrac{[\mu_a(\mathbf{r}_n)c + p_2]O(\mathbf{r}_n, p_1) - [\mu_a(\mathbf{r}_n)c + p_1]O(\mathbf{r}_n, p_2)}{p_1 - p_2} \\ \delta\mu_s'(\mathbf{r}_n) = \dfrac{[O(\mathbf{r}_n, p_1) - O(\mathbf{r}_n, p_2)]\mu_s'(\mathbf{r}_n)c}{p_1 - p_2} \end{cases}, \tag{S4}$$

During the implementation, Eqs. (S2) and (S4) were solved simultaneously within an iterative ART framework to stabilize the ill-posed inversion.[42]

S1.3 Region-wise tissue optical property estimation

Considering the limited number of measurements and the ill-posed nature of TD-DOT reconstruction, each anatomical tissue region in the personalized-scaled DB model was assumed to be optically homogeneous.[44] Therefore, node-wise reconstruction was reduced to region-wise estimation of tissue optical properties for the SS, GM, and WM regions.

The voxel-wise Jacobian matrix $\mathbf{J}$ was transformed into a region-wise Jacobian matrix using a tissue region-voxel mapping matrix:

$$\tilde{\mathbf{J}} = \mathbf{J}\mathbf{G}, \tag{S5}$$

where $\tilde{\mathbf{J}}$ represents the Jacobian matrix based on tissue regions; $\mathbf{G}$ is of size $N^{(s)}$ x C, with its term given as:

$$G_{n,c} = \begin{cases} 1 & \text{if } \mathbf{r}_n \in \Omega_c, \\ 0 & \text{otherwise} \end{cases}, \tag{S6}$$

where Ω_c is the c-th tissue region of the personalized-scaled standard-DB.

S2 TD-DOT system for in vivo data acquisition

The TD-DOT system was used for in vivo optical-property data acquisition.

S2.1 System configuration

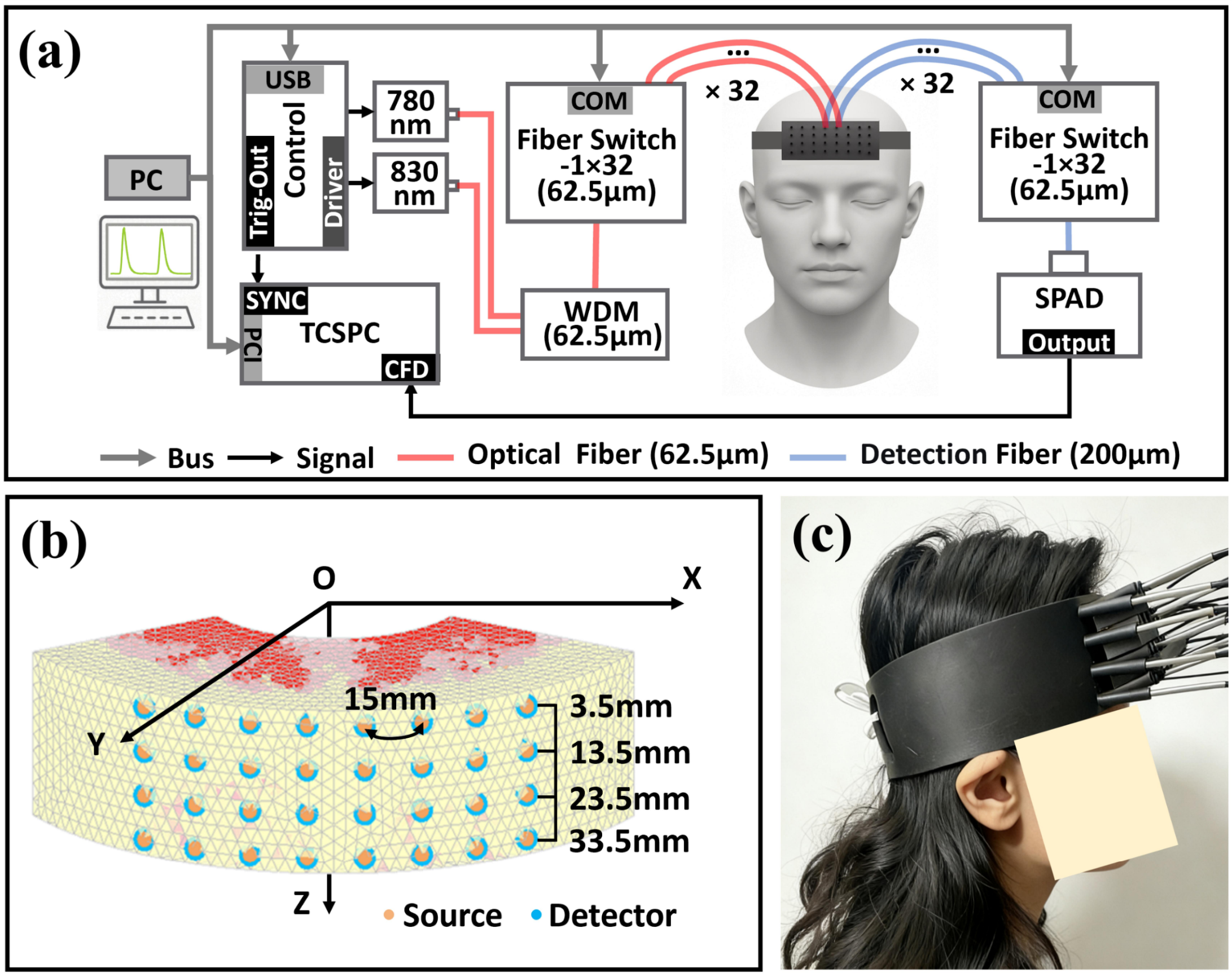

Figure S1. Dual-wavelength TD-DOT system and frontal source-detector arrangement for in vivo optical-property measurements. (a) System schematic; (b) Source-detector configuration; (c) Headband. PCI: peripheral component interconnect; USB: universal serial bus; COM: communication port; TCSPC: time-correlated single-photon counting; SYNC: synchronization signal; CFD: constant-fraction discriminator; WDM: wavelength-division multiplexer; SPAD: single-photon avalanche diode.

The TD-DOT data acquisition system is schematically illustrated in Fig. S1(a). The system supports dual-wavelength operation and incorporates adaptive integration time control. A multi-channel controller (PDL-828, PicoQuant, Germany) was used to synchronously drive

picosecond pulsed diode lasers at two wavelengths (LDH-P-780/830, PicoQuant, Germany). The two laser outputs were combined using a wavelength-division multiplexer (WDM-14P-780-830, OZ Optics, Canada), coupled into a source fiber with a core diameter of 62.5 μm and a numerical aperture of 0.22, and then distributed to individual source positions in the frontal array through a source-side optical switch (CETC-FSW1×32-MM-62.5, CETC, China). On the detection side, optical fibers with a core diameter of 200 μm and a numerical aperture of 0.22 were selected through a detection-side optical switch (CETC-FSW1×32-MM-200, CETC, China). The transmitted photons were detected by a single-photon avalanche diode (SPCM-AQRH-14-FC, Excelitas) and processed using a time-correlated single-photon counting module (TCSPC; SPC130, Becker & Hickl, Germany).

S2.2 Source-detector arrangement

The fiber arrangement over the forehead is shown in Fig. S1(b). The array consisted of 32 source positions and 32 detector positions. For each illuminated source, measurements were acquired from detectors on both sides with source-detector separations ranging from 15 to 75 mm, resulting in 200 source-detector channels. The fiber array was secured on the forehead using a black flexible headband, as shown in Fig. S1(c). The headband was positioned according to MRI anatomical landmarks, with its lower edge aligned with the superior border of the eyebrows and its lateral ends placed relative to the temples.

S2.3 Adaptive acquisition strategy

To balance signal-to-noise ratio and acquisition efficiency, an adaptive data acquisition protocol was implemented.[34] During measurement, the system monitored the TPSF peak count

for each source-detector channel and compared it with a predefined threshold of 3000 counts. Once the threshold was reached, the corresponding integration time was recorded, and the system automatically switched to the next channel. If the threshold was not reached within the maximum integration time of 3 s, the system also advanced automatically. The TCSPC time bin width was set to 12.2 ps, providing high temporal resolution for depth-sensitive measurements. Compared with fixed-integration acquisition schemes, this adaptive strategy reduced the total acquisition time while maintaining sufficient signal-to-noise ratio.

S3 MC setting

Table S1. Monte Carlo simulation parameters and optical properties assigned to different tissue layers.

Category	Parameter	Value	Description
**MC setting**	nphoton	5×10^{9}	Number of simulated photons
	tstart	0 s	Simulation starting time
	tend	5×10^{-9} s	Simulation ending time
	tstep	12.2×10^{-12} s for TPSF acquisition; 5×10^{-9} s for CW-fDOT simulation	Temporal resolution for simulation
	method	elem	Ray-tracing method
	issaveexit	1	Option to save photon exit positions and directions
	issavedet	1	Option to save detected photon information
	outputtype	flux	Output quantity
**Optical property**	SS	(0.016, 1.25) mm^{-1}	μ_a and μ_s' of the SS layer
	GM	(0.0172, 1.04) mm^{-1}	μ_a and μ_s' of the GM layer
	WM	(0.0178, 1.12) mm^{-1}	μ_a and μ_s' of the WM layer

Note: μ_a: absorption coefficient; μ_s': reduced scattering coefficient; SS: scalp/skull; GM: gray matter; WM: white matter.

MC simulations[48,49] were performed to generate forward measurement data for both time-resolved TPSF analysis and CW-fDOT reconstruction, with detailed parameters listed in Table S1. For TPSF analysis, the time step was matched to the temporal resolution of the TCSPC system. For CW-fDOT reconstruction, time-integrated fluence was used over a temporal window covering the main photon propagation period. The same MC settings were applied across different reconstruction methods to ensure that differences in the reconstructed absorption-change images mainly originated from the initialization strategies.

S4 CW-fDOT data acquisition system

As shown in Fig. S2, a self-developed portable fully parallel CDMA-fNIRS system[53] was used for CW-fDOT data acquisition. The system mainly consisted of a light source module, a detection module, a main control module, and a power supply module, enabling dual-wavelength multi-source illumination and multi-distance synchronous detection.

The light source module employed packaged dual-wavelength laser diodes (LDs; Beijing Minguan Technology Co., Ltd., China) operating at 780 and 830 nm. During data acquisition, the main control module, based on an STM32F407VET6 microcontroller (STMicroelectronics), generated CDMA-coded modulation signals and synchronously drove the LD sources through a bipolar laser diode driver integrated-circuit drive with automatic power control capability (ELM185xxb, ELMOS Semiconductor, Germany). A nonvolatile digital potentiometer (AD5259, Analog Devices, USA) was used to adjust the driving current and optical output

power. Each light source was assigned a unique pseudorandom m-sequence, allowing signals from different sources to be separated at the detection end through correlation decoding.

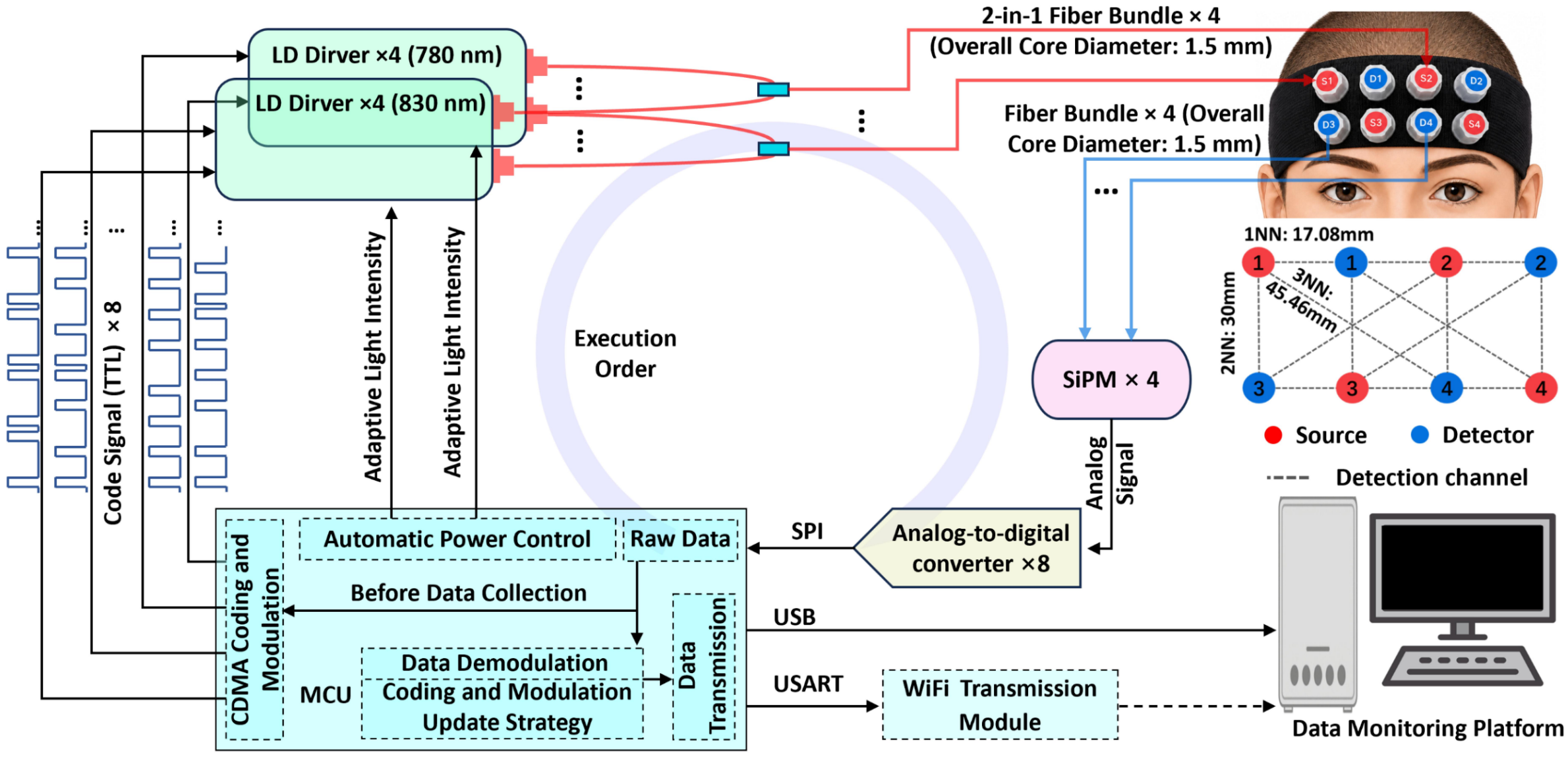

Figure S2. Portable, fully parallel CDMA-fNIRS system used for CW-fDOT acquisition. The system schematic shows the main functional modules, including CDMA coding and modulation, automatic optical-power control, dual-wavelength LD driving, fiber-bundle light delivery, SiPM detection, analog-to-digital conversion, correlation decoding, and data transmission. CDMA-fNIRS, code-division multiple-access functional near-infrared spectroscopy; CW-fDOT, continuous-wave functional diffuse optical tomography; LD, laser diode; MCU, microcontroller unit; SiPM, silicon photomultiplier; SPI, serial peripheral interface; USB, universal serial bus; USART, universal synchronous/asynchronous receiver/transmitter; TTL, transistor-transistor logic.

The dual-wavelength light output was coupled into a combined optical fiber bundle and delivered to the scalp surface. The fiber bundle consisted of multiple multimode fibers, with a single-fiber core diameter of 50 μm, a numerical aperture of 0.22, and an overall bundle core diameter of 1.5 mm. The optode array adopted an interleaved source-detector configuration

with four sources and four detectors. According to the array geometry and valid source-detector combinations, 14 measurement channels were formed, including six 1NN channels with a source-detector distance of 17.08 mm, four 2NN channels with a distance of 30.00 mm, and four 3NN channels with a distance of 45.46 mm.

After undergoing absorption and scattering within the tissue, the remitted light was detected at the scalp surface by J-Series 30035 silicon photomultipliers (SiPMs; onsemi, USA) and converted into electrical signals. The analog signals were then digitized by a 16-bit, eight-channel analog-to-digital converter (AD7606, Analog Devices, USA) and transmitted to the main control module for correlation decoding, signal separation, and data transfer. The power supply module consisted of two 18650 lithium batteries and an isolated 5 V/60 W DC–DC buck converter (GOD60-24S05B3C, GODSEND).

S5 Dual-target numerical simulation

To evaluate the ability to resolve spatially separated activations, a dual-target numerical simulation was performed using the same background optical properties, absorption contrasts (1.5, 2, 3), and source-detector configuration as the preceding single-target simulation. As shown in Fig. S3(a), two spherical targets with a radius of 7.5 mm were placed at (±21.22, 68.8, 18.5) mm within the standard DB model. Light propagation was simulated using MC, and absorption-change $\Delta\mu_a$ maps were reconstructed using the Homo-Emp. init. and SOB init. methods. As shown in Fig. S3(b), SOB init. clearly separated the two targets, with reconstructed locations and contours closely matching the ground truth. In contrast, Homo-Emp. init. showed more pronounced shape distortion, boundary spreading, and reduced target separability. The cross-sectional profiles further confirmed that SOB init. more accurately

recovered the target peak locations and bifocal morphology across different absorption contrasts. Quantitative results in Fig. S3(c) showed that SOB init. consistently achieved lower MAE, higher SSIM, and higher CNR than Homo-Emp. init. These results demonstrate that SOB init. maintained superior reconstruction accuracy, structural fidelity, and contrast-to-noise performance in dual-target reconstruction.

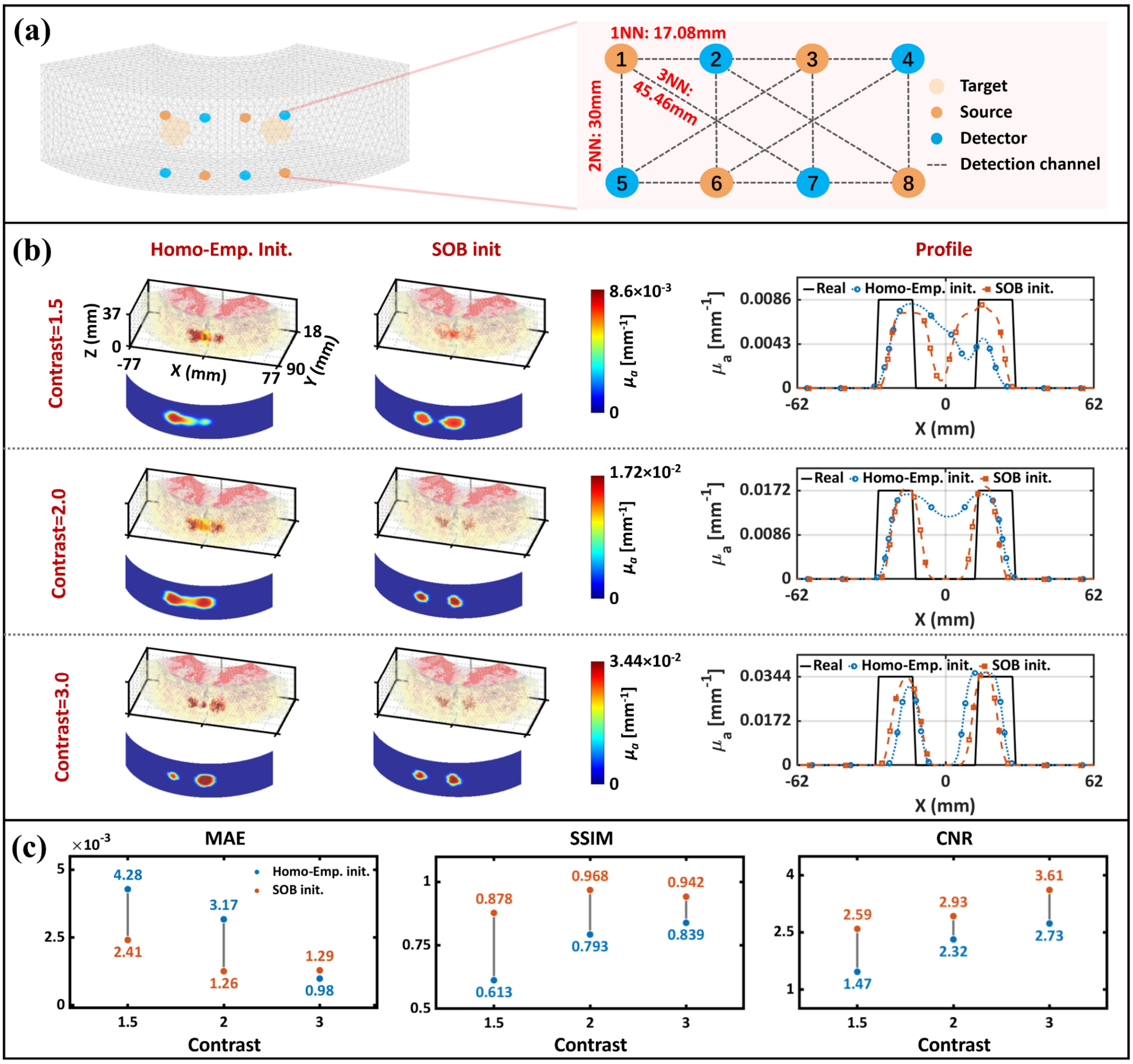

Figure S3. Dual-target numerical reconstruction under three absorption-contrast conditions. (a) Numerical model containing two spherical targets of 7.5-mm radius centered at (−21.22, 68.8, 18.5) mm and (21.22, 68.8, 18.5) mm, together with the interleaved four-source/four-detector array. (b) Reconstructed 3D images, 2D maps, and

cross-sectional profiles obtained using Homo-Emp. init. and SOB init. (c) Quantitative evaluation of dual-target reconstruction using MAE, SSIM, and CNR.

S6 SS-layer interference effects on GM-layer reconstruction

In practical CW-fDOT measurements, boundary signals reflect both GM task-related hemodynamic responses and SS-layer physiological fluctuations,[1-3] which may cause amplitude bias, spatial spreading, or localization shifts in GM-layer reconstruction.[59] To evaluate the robustness of the proposed SOB-guided distance-dependent CW-fDOT framework, two conditions were compared under identical forward-model, optical-property, and reconstruction settings: GM activation only (Set A) and GM activation with SS interference (Set B). Set A included the hemodynamic response function (HRF) only in the GM target region, whereas Set B used the same GM HRF with additional SS-layer physiological interference. The reconstructed GM results were compared to assess the effects of SS-layer interference on GM activation localization, morphology, and amplitude.

S6.1 Simulation design

Numerical simulations used the same DB geometry, tissue optical properties, source-detector configuration, and MC settings (Table S1) as the single-target simulation. As shown in Fig. S4(a), a spherical activation target with a radius of 7.5 mm was placed at x=0 mm, y=72 mm, and z=18.5 mm to simulate a localized GM activation. Task-related hemodynamic changes were applied only within the GM target region. SS-layer physiological interference was added only in Set B. The corresponding absorption changes were defined as follows:[59]

$$\begin{cases} \delta\mu_a^{\mathrm{GM}}(t,\lambda) = \delta\mu_C^{\mathrm{GM}}(t,\lambda), \\ \delta\mu_a^{\mathrm{SS}}(t) = \varphi\,\delta\tilde{\mu}_P^{\mathrm{SS}}(t). \end{cases} \tag{S7}$$

where, $\delta\mu_a^{\mathrm{GM}}(t)$ and $\delta\mu_a^{\mathrm{SS}}(t)$ denote the absorption changes in the GM activation region and SS layer, respectively. $\delta\mu_C^{\mathrm{GM}}(t,\lambda)$ denotes the GM absorption change induced by the task-related hemodynamic response, and $\delta\tilde{\mu}_P^{\mathrm{SS}}(t)$ denotes the absorption change induced by SS physiological interference. To prevent the physiological component from dominating the absorption perturbation because of its larger variance, a scaling factor $\varphi = \sigma_C/\sigma_P$ was introduced, where σ_C and σ_P are the standard deviations of the GM activation response and the original physiological interference, respectively.

S6.1.1 GM-layer hemodynamic activity

The GM-layer absorption change was modeled as the product of a spatial activation mask, a wavelength-dependent absorption scaling factor, and a task-related temporal response:

$$\delta\mu_C(t,\lambda) = \mathbf{A}[s(\lambda) \odot c(t)], \tag{S8}$$

where $\mathbf{A}$ is a binary spatial activation mask. $A_{\boldsymbol{n}} = 1$ for nodes inside the target activation region and $A_{\boldsymbol{n}} = 0$ otherwise; n denotes the n-th node. The wavelength-dependent scaling factor $s(\lambda)$ was defined as:

$$s(\lambda) = \xi\varepsilon_{\mathrm{HbO}}(\lambda) - (1-\xi)\varepsilon_{\mathrm{HbR}}(\lambda), \tag{S9}$$

where $\varepsilon_{\mathrm{HbO}}(\lambda)$ and $\varepsilon_{\mathrm{HbR}}(\lambda)$ are the molar extinction coefficients of HbO and HbR, respectively. In this study, $\varepsilon_{\mathrm{HbO}}(\lambda) = 0.71\ \mathrm{mM^{-1}\cdot cm^{-1}}$, $\varepsilon_{\mathrm{HbR}}(\lambda) = 1.08\ \mathrm{mM^{-1}\cdot cm^{-1}}$, and $\xi = 0.7$ was used to weight the HbO change relative to the HbR change.

The temporal response $c(t)$ was obtained by convolving the task sequence $x(t)$ with the hemodynamic response function $\mathrm{HRF}(t;\kappa,\tau,\omega)$:

$$c(t) = HRF(t, \kappa, \tau, \omega) * x(t), \tag{S10}$$

The HRF was modeled using a standard double-gamma function:

$$HRF(t, \kappa, \tau, \omega) = \kappa \left[\frac{t^{\tau-1}\omega^{\tau}e^{-\omega t}}{\Gamma(\tau)} - \frac{1}{6}\frac{t^{\tau+\tau_0-1}\omega^{\tau+\tau_0}e^{-\omega t}}{\Gamma(\tau+\tau_0)}\right], \tag{S11}$$

The task sequence matched the in vivo paradigm, including a 30 s resting baseline, a 30 s task period, and a 30 s post-task recovery period:

$$\boldsymbol{x}(t) = \begin{cases} 1, & 30 \leq t < 60 \text{ s} \\ 0, & 0 \leq t < 30 \text{ s or } 60 \leq t < 90 \text{ s} \end{cases}, \tag{S12}$$

where κ, τ, and ω control the HRF amplitude, peak latency, and temporal width, respectively, and τ_0 controls the delay between the positive peak and the subsequent undershoot. In this study, $\kappa = 0.0258 \text{ mm}^{-1}$, τ was randomly sampled from 4-10 s, ω was randomly sampled from 1.6-3.3 s, and $\tau_0 = 10$ s. The task sequence was sampled at 20 Hz, consistent with the CW-fDOT acquisition frequency.

S6.1.2 SS-layer physiological interference

SS-layer physiological interference was generated in the frequency domain and then converted into a TD absorption perturbation using the inverse Fourier transform. A complex spectrum with random phases was constructed from a predefined power spectral density (PSD):

$$\delta\tilde{\mu}_P^{\text{SS}}(t) = \mathcal{F}^{-1}[\delta M_P(f)], \tag{S13}$$

where the complex frequency-domain signal was defined as:

$$\delta M_P(f) = \sqrt{\text{PSD}(f)\frac{df}{2}}e^{j\alpha(f)}, \tag{S14}$$

where, f is the frequency, $\mathcal{F}^{-1}$ denotes the inverse Fourier transform, df is the frequency resolution, j is the imaginary unit, and $\alpha(f)$ is a randomly sampled phase. To simulate superficial physiological rhythms, the PSD was modeled as the sum of three Gaussian peaks representing Mayer waves, respiration, and cardiac activity:

$$\mathrm{PSD}(f) = \sum_{b=1}^{3} \frac{\alpha_b}{\sqrt{2\pi}\sigma_b} \exp\left[-\frac{1}{2}\left(\frac{f-\nu_b}{\sigma_b}\right)^2\right], \tag{S15}$$

where, b denotes the physiological rhythm component, and α_b, ν_b, and σ_b represent the amplitude weight, center frequency, and spectral width of the bth component, respectively. In this study, the amplitude weights of Mayer waves, respiration, and cardiac activity were set to 1.00, 0.70, and 0.50, respectively; the corresponding center frequencies were 0.10, 0.30, and 1.10 Hz; and the corresponding spectral widths were 0.015, 0.020, and 0.080 Hz.

S6.2 Results

Fig. S4(b) shows the temporal variations in the mean absorption coefficients of the SS, GM, and WM layers and the active region under conditions with and without SS-layer physiological interference. As shown in Fig. S4(c), after SS-layer physiological interference was introduced, the reconstructed profile at 35 s, corresponding to the rising phase of the GM response, showed only slight lateral broadening and weak side fluctuations, while the main peak remained near the true target. At 50 s, when the GM response was stronger, the reconstructed target became more concentrated, the transverse profile more closely matched the true target contour, and no obvious spatial shift was observed. The quantitative results in Fig. S4(d) showed that SS-layer interference caused only limited performance degradation. At 35 s, MAE increased by approximately 8.45%, while SSIM and CNR decreased by 3.71% and 2.18%, respectively. At

50 s, MAE increased by 48.89%, mainly because the MAE without physiological interference was relatively small, whereas SSIM and CNR decreased by only 2.11% and 2.72%, respectively. Overall, SS-layer interference mainly increased the reconstruction error, while its effects on spatial similarity and contrast-to-noise performance were limited, without causing localization failure or obvious morphological distortion. These results indicate that, under the current simulated interference condition, the proposed SOB-guided distance-dependent CW-fDOT reconstruction framework remained able to recover the dominant GM activation response from mixed boundary measurements and exhibited robustness to superficial physiological interference.

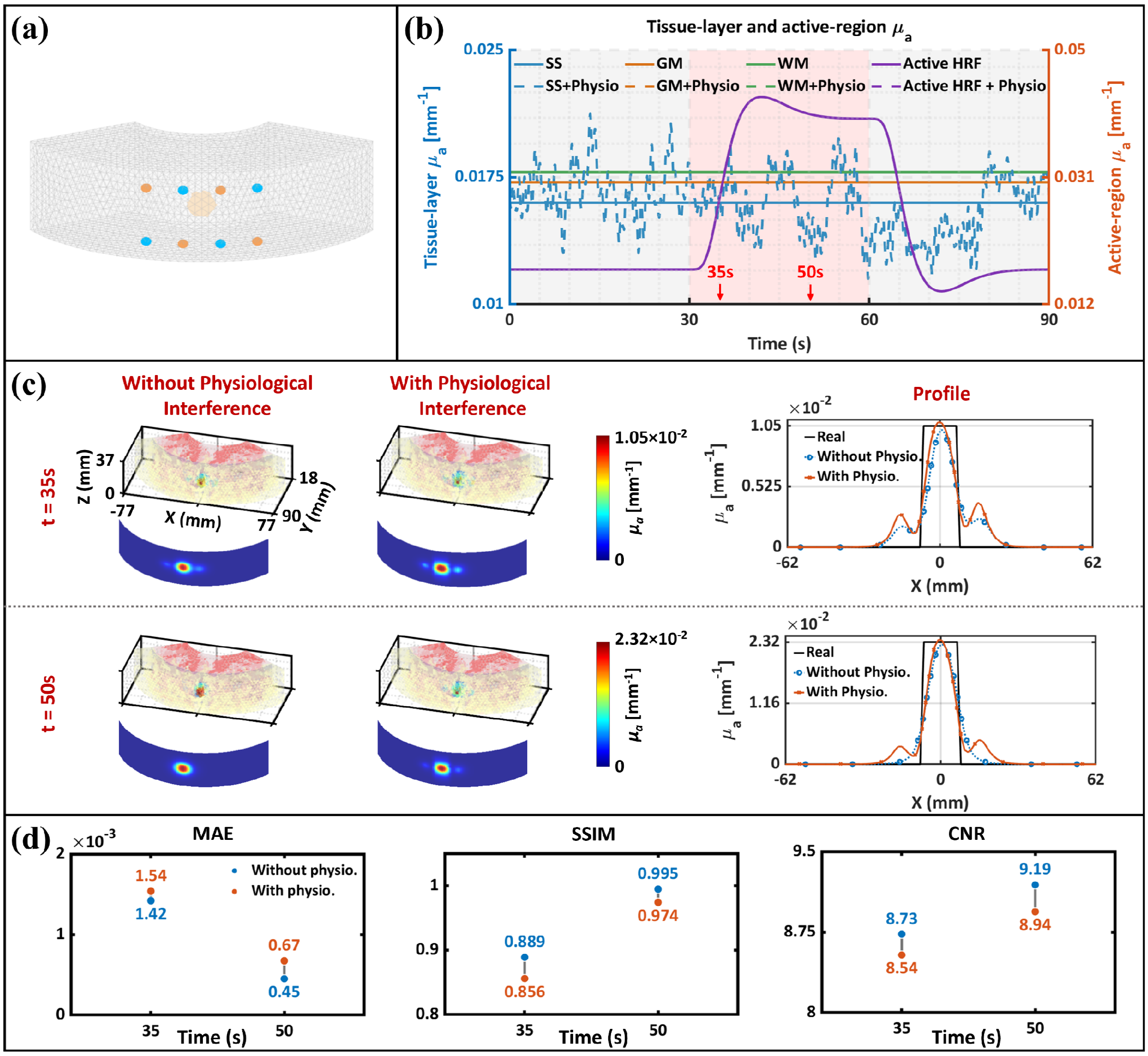

Figure S4. SS-layer interference effects on GM-layer reconstruction. (a) Source-detector arrangement and spherical GM activation target. (b) Temporal variations in the mean absorption coefficients of the SS, GM, and WM layers and the active region under conditions with and without SS-layer physiological interference. Red arrows indicate the reconstruction time points at 35 and 50 s. (c) Comparison of GM-layer 3D reconstructions, 2D maps, and cross-sectional profiles with and without SS-layer physiological interference at 35 and 50 s. (d) Quantitative evaluation of GM-layer reconstruction using MAE, SSIM, and CNR.
